# The ApiAP2 factor PfAP2-HC is an integral component of heterochromatin in the malaria parasite *Plasmodium falciparum*

**DOI:** 10.1016/j.isci.2021.102444

**Published:** 2021-04-16

**Authors:** Eilidh Carrington, Roel Henrikus Martinus Cooijmans, Dominique Keller, Christa Geeke Toenhake, Richárd Bártfai, Till Steffen Voss

**Affiliations:** 1 Department of Medical Parasitology and Infection Biology, Swiss Tropical and Public Health Institute, 4051 Basel, Switzerland; 2 University of Basel, 4001 Basel, Switzerland; 3 Department of Molecular Biology, Radboud University, 6525GA Nijmegen, The Netherlands

**Keywords:** Parasitology, Cell Biology

## Abstract

Malaria parasites undergo a complex life cycle in the human host and the mosquito vector. The ApiAP2 family of DNA-binding proteins plays a dominant role in parasite development and life cycle progression. Most ApiAP2 factors studied to date act as transcription factors regulating stage-specific gene expression. Here, we characterized an ApiAP2 factor in *Plasmodium falciparum* that we termed PfAP2-HC. We demonstrate that PfAP2-HC specifically binds to heterochromatin throughout the genome. Intriguingly, PfAP2-HC does not bind DNA *in vivo* and recruitment of PfAP2-HC to heterochromatin is independent of its DNA-binding domain but strictly dependent on heterochromatin protein 1. Furthermore, our results suggest that PfAP2-HC functions neither in the regulation of gene expression nor in heterochromatin formation or maintenance. In summary, our findings reveal PfAP2-HC as a core component of heterochromatin in malaria parasites and identify unexpected properties and substantial functional divergence among the members of the ApiAP2 family of regulatory proteins.

## Highlights

• The ApiAP2 factor AP2-HC is a core component of heterochromatin in malaria parasites
• Binding of AP2-HC to heterochromatin strictly depends on heterochromatin protein 1
• The AP2 DNA-binding domain of AP2-HC is dispensable for heterochromatin association

## Graphical abstract

**Figure:**
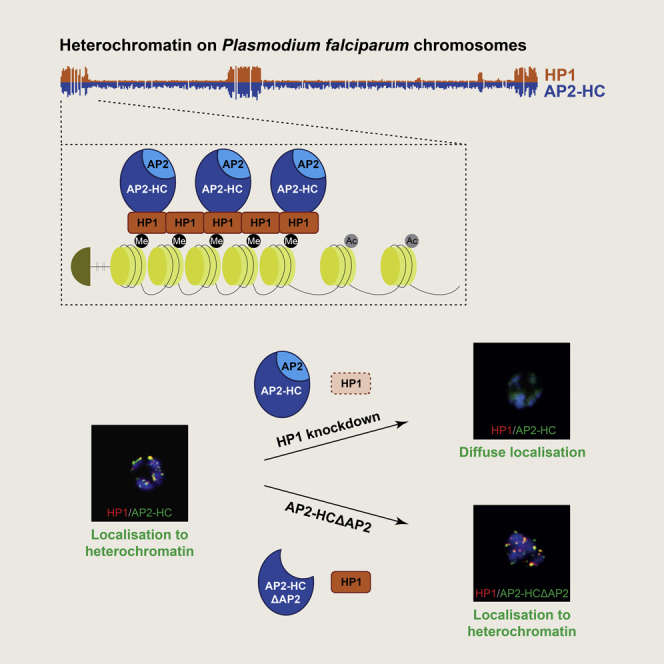

## Introduction

The apicomplexan parasite *Plasmodium falciparum* is the main cause of severe malaria worldwide, with the majority of the estimated 405,000 malarial deaths in 2018 attributed to this pathogen (WHO, 2019). The symptoms of the disease occur owing to repeated asexual intraerythrocytic developmental cycles (IDCs), where merozoite stage parasites invade human red blood cells (RBCs) and develop through the ring stage (0–24 h post invasion [hpi]) and trophozoite stage (24–30 hpi), before undergoing schizogony to produce mature segmented schizonts containing up to 32 merozoites (30–48 hpi). Rupture of the infected RBCs (iRBCs) releases the merozoites, which in turn undergo another IDC after invading new RBCs. A small proportion of schizonts per cycle commit to the sexual development pathway and produce ring stage daughter cells that mature over a period of 10 days and through four intermediate stages (I–IV) into mature stage V gametocytes (Venugopal et al., 2020). Circulating stage V gametocytes are the only forms of the parasite able to infect the mosquito vector and are therefore essential for malaria transmission.

A key trait of *P. falciparum* is the ability to adapt to and evade the constantly changing environment in its human host through clonally variant gene expression, a process vital to a broad range of biological processes, including antigenic variation, RBC invasion, solute transport, and sexual conversion (Duraisingh and Skillman, 2018; Llora-Batlle et al., 2019; Rovira-Graells et al., 2012). Clonally variant gene expression in *P. falciparum* is regulated epigenetically, with heritable gene silencing mediated by heterochromatin (Voss et al., 2014). Heterochromatin is found at subtelomeric regions on all 14 chromosomes and in some chromosome internal islands and is characterized by the binding of heterochromatin protein 1 (PfHP1) to the histone modification histone 3 lysine 9 trimethylation (H3K9me3) (Flueck et al., 2009; Fraschka et al., 2018; Lopez-Rubio et al., 2009; Perez-Toledo et al., 2009; Salcedo-Amaya et al., 2009). These PfHP1/H3K9me3-demarcated heterochromatic domains cover over 400 genes in total (approximately 8% of all protein-coding genes in the genome) (Flueck et al., 2009; Fraschka et al., 2018). As a core component of heterochromatin, PfHP1 plays an essential role in heterochromatic gene silencing and has a multi-faceted role in parasite biology as previously demonstrated with a conditional loss-of-function mutant (Brancucci et al., 2014). Conditional depletion of PfHP1 resulted in the de-repression of multi-copy gene families important in antigenic variation, including the well-characterized *var* gene family (Brancucci et al., 2014; Scherf et al., 2008). In addition, around half of progeny parasites depleted of PfHP1 underwent gametocytogenesis due to de-repression of the internal heterochromatic *pfap2-g* locus encoding the master transcriptional regulator of gametocytogenesis, PfAP2-G (Brancucci et al., 2014; Kafsack et al., 2014; Sinha et al., 2014). The remaining progeny arrested at the trophozoite stage, indicating an essential role of PfHP1 in proliferation (Brancucci et al., 2014). With such a diverse range of processes reliant on PfHP1 and heterochromatic gene silencing, the mechanisms of this system warrant further study. However, the molecular machinery involved in heterochromatin establishment, spreading, and maintenance in *P. falciparum* remain elusive, along with the transcription factors involved in regulating the expression of heterochromatic genes.

The main transcription factor family in Apicomplexan parasites is the ApiAP2 group of DNA-binding proteins, comprising 27 members in *P. falciparum* (Balaji et al., 2005; Jeninga et al., 2019; Painter et al., 2011). ApiAP2 proteins are characterized by the presence of one to three AP2 domains, homologous to the DNA-binding domains of plant APETALA2/ethylene response element binding protein (AP2/EREBP) transcription factors (Balaji et al., 2005; Dietz et al., 2010). To date, five members have been functionally analyzed in *P. falciparum*, three of which are acting as transcription factors. PfAP2-G, as mentioned above, is the master regulator of sexual commitment (Kafsack et al., 2014) and has recently been confirmed as an activator of gametocyte genes, with an additional role in regulating RBC invasion genes suggested (Josling et al., 2020). PfAP2-I is likely essential for parasite survival and regulates a subset of gene families involved in RBC invasion (Santos et al., 2017). Of interest, PfAP2-I and PfAP2-G also bind upstream of several genes encoding ApiAP2 factors, which could suggest a complex regulatory interplay between ApiAP2 family members (Josling et al., 2020; Santos et al., 2017). Indeed, Josling and colleagues provided evidence of cooperative binding of PfAP2-G and PfAP2-I to some invasion gene promoters (Josling et al., 2020). PfAP2-EXP is involved in regulating multi-gene families, including *rif*, *stevor*, and *pfmc-2tm*, and is seemingly essential for asexual growth (Martins et al., 2017). In contrast, PfSIP2 predominantly binds to SPE2 motifs found in telomere-associated repeat elements (TAREs) and upstream of subtelomeric upsB *var* genes, both of which are heterochromatic, suggesting a possible role in heterochromatin and/or chromosome end biology (Flueck et al., 2010). Finally, PfAP2-Tel binds to telomere repeats on all 14 chromosomes and is likely involved in telomere maintenance mechanisms (Sierra-Miranda et al., 2017). Beyond these studies in *P. falciparum*, much has been achieved in characterizing ApiAP2 proteins of *Plasmodium* species infecting rodents. In *P. berghei*, several ApiAP2 factors with essential roles in gametocytogenesis (Sinha et al., 2014; Yuda et al., 2015, 2020) and in the mosquito and liver stages (Iwanaga et al., 2012; Kaneko et al., 2015; Yuda et al., 2009, 2010) have been studied. In addition, systematic knockout screens in *P. berghei* (Modrzynska et al., 2017) and *P. yoelii* (Zhang et al., 2017) provided an extensive characterization of the ApiAP2 family and highlight essentiality at different life cycle stages. Of interest, although some orthologs have the same function in *P. falciparum* and *P. berghei*, for example, AP2-G (Kafsack et al., 2014; Sinha et al., 2014), others display differences such as the PfAP2-EXP ortholog PbAP2-SP, which is expressed exclusively in the sporozoite stages of *P. berghei* (Yuda et al., 2010).

We recently identified the ApiAP2 protein PF3D7_1456000 as a putative interaction partner of PfHP1 using co-immunoprecipitation (coIP) experiments coupled with protein mass spectrometry (Filarsky et al., 2018). Here, we present a multifaceted approach to dissect the potential functions of this ApiAP2 factor in heterochromatin-associated processes during blood stage development of *P. falciparum* parasites.

## Results

### PfAP2-HC specifically associates with heterochromatin

**Figure 1:**
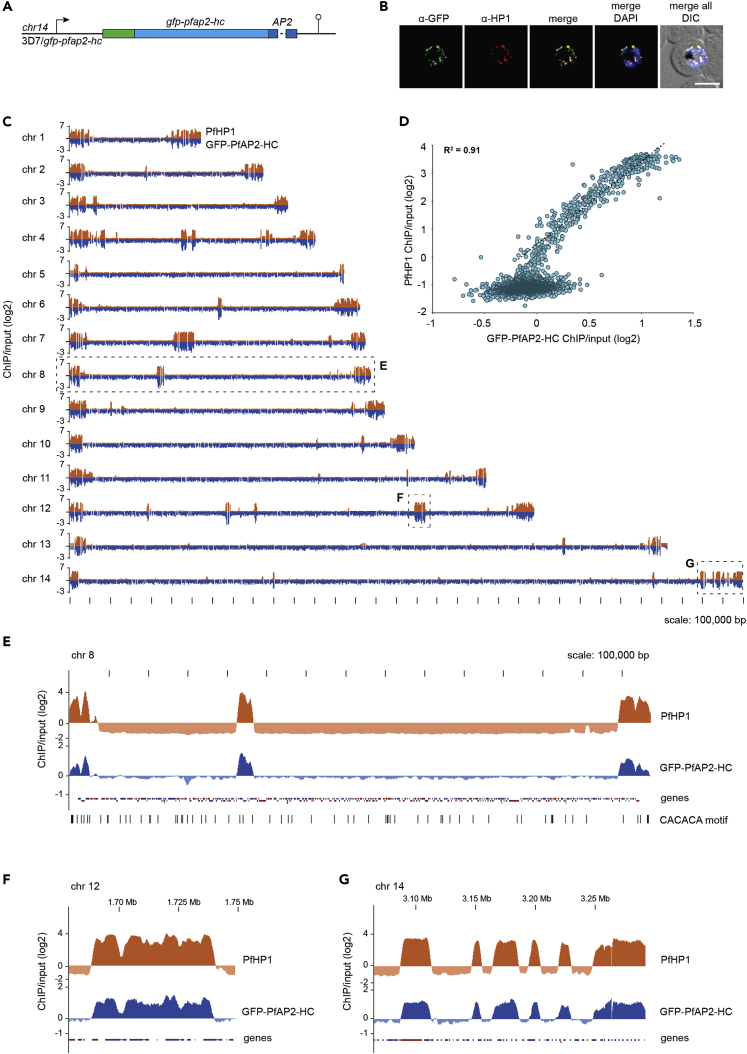
PfAP2-HC associates with PfHP1 throughout the genome (A) Schematic map of the endogenous *pfap2-hc* locus after introduction of a *gfp* tag by CRISPR-Cas9-mediated gene editing in 3D7/GFP-PfAP2-HC parasites. See also Figure S1. (B) Representative IFA images of GFP-PfAP2-HC and PfHP1 localization in 3D7/GFP-PfAP2-HC parasites, 36–44 hpi. Nuclei were stained with DAPI. DIC, differential interference contrast. Scale bar, 5 μm. (C) Log2-transformed α-PfHP1 (orange) and α-GFP (blue) ChIP-over-input ratio tracks obtained from 3D7/GFP-PfAP2-HC schizont stage parasites. α-PfHP1 and α-GFP ChIP tracks have been offset by 2 and 1, respectively, to be able to display the full scale of variation. In addition, α-GFP ChIP-seq data are mirrored on a negative scale. Dashed boxes highlight regions that are enlarged in (E)–(G). (D) Scatterplot of average log2-transformed α-PfHP1 and α-GFP ChIP-over-input values for all parasite genes. The depicted regression line is based on heterochromatic genes only (log2 ratio α-PfHP1/input ≥0). The coefficient of determination (R^2^) is displayed in the upper left corner. See also Data S1. (E) Log2-transformed ChIP-over-input ratio tracks on chromosome 8. The locations of the putative PfAP2-HC binding motif (CACACA) (Campbell et al., 2010) are shown below the tracks (FDR <0.05). Coding sequences are shown as blue (sense strand) and red (antisense strand) boxes. (F and G) Log2-transformed ChIP-over-input ratio tracks over sections of chromosome 12 and 14, respectively.

We recently identified a list of potential PfHP1 interaction partners, which includes a member of the ApiAP2 family of putative transcription factors, PF3D7_1456000 (Filarsky et al., 2018), hereafter referred to as PfAP2-HC. To validate the interaction between PfAP2-HC and PfHP1, we employed a two-plasmid CRISPR-Cas9-based gene editing approach to N-terminally tag PfAP2-HC with GFP (GFP-PfAP2-HC) (Figures 1A and S1). We tagged the N terminus because the single AP2 domain of PfAP2-HC is located right at the C terminus of the protein where tagging may interfere with its function. We obtained a clonal line of the resulting 3D7/GFP-PfAP2-HC population by limiting dilution cloning and confirmed correct editing of the locus by PCR on genomic DNA (gDNA) (Figure S1). Live cell fluorescence imaging of GFP-PfAP2-HC revealed a perinuclear localization, which was undetectable in ring stages and first appeared in trophozoites, peaking mid-schizogony and decreasing in late schizonts (Figure S1). This temporal expression pattern is consistent with the transcriptional profile of *pfap2-hc* during the IDC (Bartfai et al., 2010). The localization pattern of GFP-PfAP2-HC matches that of PfHP1 in immunofluorescence assays (IFAs), where the two proteins appear to overlap (Figure 1B).

In order to investigate the genome-wide binding profile of GFP-PfAP2-HC and to allow comparison with PfHP1 at high resolution, we performed chromatin immunoprecipitation-sequencing (ChIP-seq) using α-GFP and α-PfHP1 antibodies to compare binding profiles within the same parasite population. We found that GFP-PfAP2-HC indeed co-localizes with PfHP1 throughout the genome (Figures 1C, 1E, 1F and 1G). To quantify the degree of co-localization, we computed and compared PfHP1 and PfAP2-HC ChIP-over-input enrichment values in coding regions across the genome (Data S1). This confirmed a strong correlation (R^2^ = 0.91) between PfAP2-HC and PfHP1 occupancies across coding regions of all heterochromatic genes (Figure 1D). In addition, we visualized on all chromosomes the locations of the putative PfAP2-HC target DNA motif (CACACA) as predicted by *in vitro* binding preference of the recombinant PfAP2-HC AP2 DBD (Campbell et al., 2010). The CACACA motif showed no enrichment in heterochromatic over euchromatic regions and therefore showed no positional association with the *in vivo* PfAP2-HC binding profile (Figure 1E). Collectively, these findings show that PfAP2-HC localizes exclusively to PfHP1-defined heterochromatic regions and seems not to bind to the predicted CACACA target motifs *in vivo*.

### PfAP2-HC is not required for heterochromatin maintenance and inheritance

**Figure 2:**
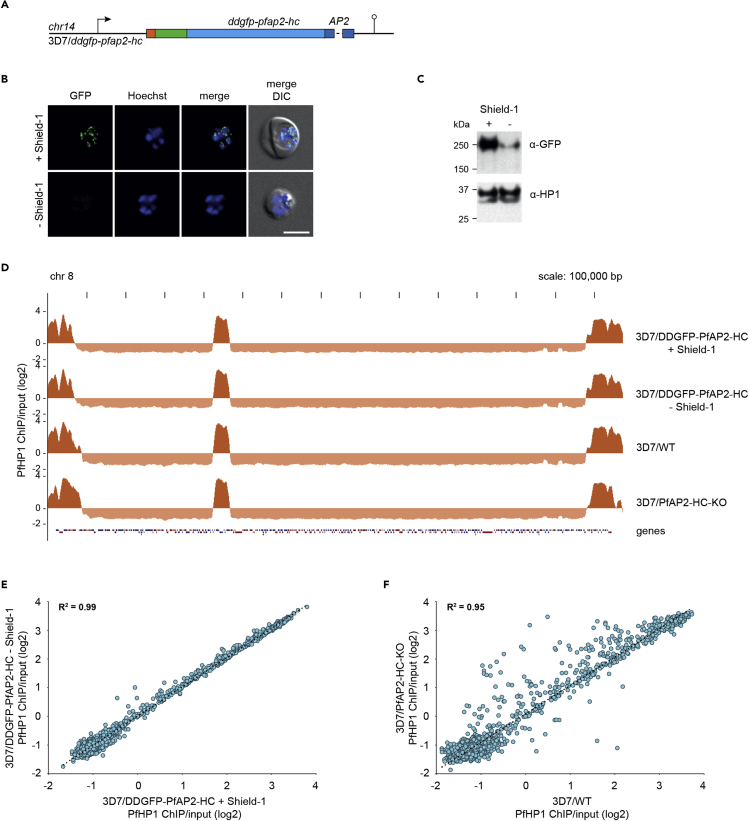
PfAP2-HC depletion does not affect PfHP1 genome-wide coverage (A) Schematic map of the endogenous *pfap2-hc* locus after CRISPR-Cas9-mediated gene editing to introduce *ddgfp* tag in 3D7/DDGFP-PfAP2-HC parasites. See also Figure S2. (B) Representative live cell fluorescence images of 3D7/DDGFP-PfAP2-HC schizonts (36–44 hpi) grown in the presence (+) or absence (−) of Shield-1. Nuclei were stained with Hoechst. DIC, differential interference contrast. Scale bar, 5 μm. See also Figure S3. (C) Western blot showing DDGFP-PfAP2-HC expression levels in 3D7/DDGFP-PfAP2-HC schizonts (36–44 hpi) grown in the presence (+) or absence (−) of Shield-1. PfHP1 expression levels served as a loading control. The full-sized blot is available in Figure S2. (D) Log2-transformed α-PfHP1 ChIP-over-input tracks from 3D7/DDGFP-PfAP2-HC schizont stage parasites grown in the presence (+) or absence (−) of Shield-1 (top two tracks). Log2-transformed α-PfHP1 ChIP-over-input tracks from 3D7/WT and 3D7/PfAP2-HC-KO schizonts (bottom two tracks). Coding sequences are shown as blue (sense strand) and red (antisense strand) boxes. See also Figure S4. (E and F) Scatterplots of average log2-transformed α-PfHP1 ChIP-over-input values at all coding regions in 3D7/DDGFP-PfAP2-HC schizonts grown in the presence (+) or absence (−) of Shield-1 (E) and in 3D7/WT and 3D7/PfAP2-HC-KO schizonts (F). Depicted regression lines are based on heterochromatic genes only (log2 ratio α-PfHP1/input ≥0). The coefficient of determination (R^2^) is shown in the upper left corner. See also Data S1.

Having shown that PfAP2-HC shares the genome-wide binding profile of PfHP1, we next investigated the function of this ApiAP2 factor by creating a conditional knockdown line employing the FKBP destabilization domain (DD) system. DD-tagged proteins are stabilized in the presence of the small molecule Shield-1, and removal of this ligand leads to protein degradation (Armstrong and Goldberg, 2007; Banaszynski et al., 2006). We utilized our two-plasmid CRISPR-Cas9 approach to N-terminally tag PfAP2-HC with DDGFP to create the cell line 3D7/DDGFP-PfAP2-HC (Figures 2A and S2). Limiting dilution cloning resulted in a parasite clone containing the correctly edited locus, which we confirmed by PCR on gDNA (Figure S2). Substantial depletion of DDGFP-PfAP2-HC expression in the absence of Shield-1 was verified by live cell fluorescence imaging (Figure 2B) and Western blot (Figures 2C and S2). Depletion of DDGFP-PfAP2-HC expression caused no major cell cycle- or proliferation-related phenotypes nor did it have an effect on sexual conversion rates (Figure S3).

In order to investigate the potential effect of PfAP2-HC depletion on heterochromatin, we grew parasites in the presence or absence of Shield-1 for 13 generations and compared their genome-wide PfHP1 binding profiles by ChIP-seq. The genome-wide PfHP1 coverage tracks in 3D7/DDGFP-PfAP2-HC parasites grown in the absence or presence of Shield-1 are highly similar (Figure 2D). Likewise, the genome-wide PfHP1 coverage of coding regions in the two populations is nearly identical (R^2^ = 0.99) (Figure 2E and Data S1) showing that depletion of PfAP2-HC has no discernible effect on PfHP1 localization on chromatin. To test whether the lack of obvious loss-of-function phenotypes was due to the residual amounts of DDGFP-PfAP2-HC protein remaining after Shield-1 removal (Figure 2C), we also generated a PfAP2-HC knockout cell line, 3D7/PfAP2-HC-KO (Figure S4), which we confirmed by PCR on gDNA (Figure S4). 3D7/PfAP2-HC-KO parasites did not show obvious growth-related phenotypic changes either (Figure S4) and maintained PfHP1 occupancy at levels similar to 3D7 wildtype (3D7/WT) and 3D7/DDGFP-PfAP2-HC parasites (Figure 2D). Changes in PfHP1 coverage of some genes were observed in 3D7/PfAP2-HC-KO parasites compared with 3D7/WT and 3D7/DDGFP-PfAP2-HC (Figures 2F and S4 and Data S1). However, these changes are likely unrelated to the lack of PfAP2-HC expression but rather attributable to clonally variant changes in PfHP1 occupancy as similar differences are observed when comparing different PfAP2-HC-expressing clonal lines (3D7/WT and 3D7/DDGFP-PfAP2-HC) (Figure S4). Together, these results show that PfAP2-HC is neither required for asexual proliferation nor for the maintenance and inheritance of PfHP1-demarcated heterochromatin.

### PfAP2-HC does not act as a transcription factor in blood stage parasites

**Figure 3:**
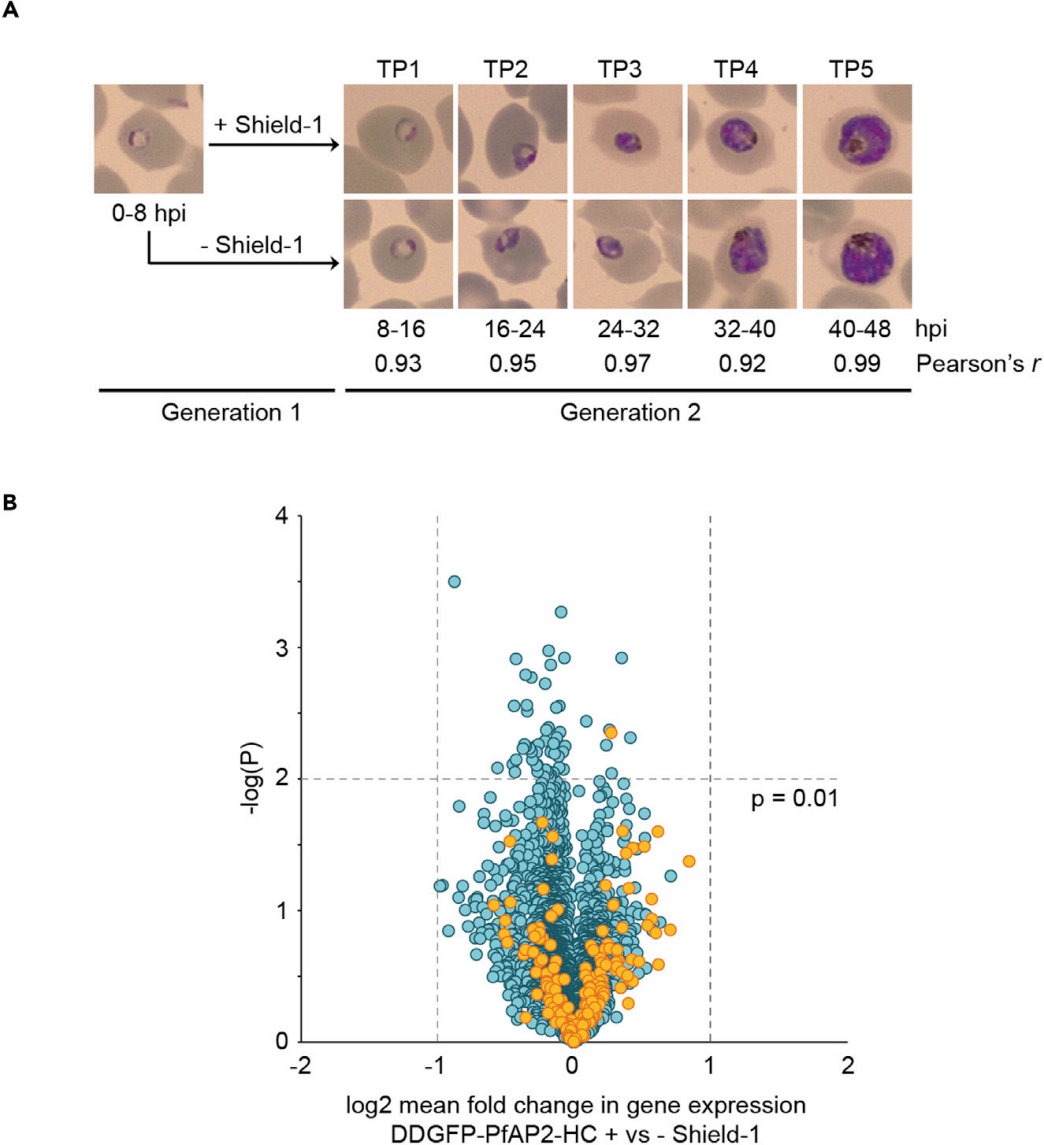
Depletion of PfAP2-HC has no effect on transcription in asexual blood stage parasites (A) 3D7/DDGFP-PfAP2-HC parasites were grown in the presence (+) or absence (−) of Shield-1 from 0–8 hpi for one generation and sampled for comparative transcriptome analysis at five IDC time points in the subsequent generation. Pearson correlation coefficients *r* indicate the pairwise correlation between the paired transcriptomes of parasites cultured in the presence (+) or absence (−) of Shield-1 for each time point. TP, time point. See also Data S2. (B) Volcano plot showing log2 fold changes in relative transcript abundance averaged across the five time points and plotted against significance [-log10(p value)]. Euchromatic and heterochromatic genes are depicted by blue and orange circles, respectively. See also Data S2.

To identify any possible role of PfAP2-HC in transcriptional regulation we performed a transcriptome-wide microarray time course analysis. We compared 3D7/DDGFP-PfAP2-HC parasites grown in the presence and absence of Shield-1 across five time points throughout the IDC (Figure 3A). For each of the five time points the paired transcriptome data were strongly correlated based on Pearson correlation values, demonstrating highly comparable stage composition across the time course (Figure 3A and Data S2). We found no significant difference in gene expression, with no transcripts showing greater than 2-fold average fold change in steady-state mRNA abundance between the +Shield-1 and -Shield-1 populations (Figure 3B), suggesting that PfAP2-HC does not play a dominant role in transcriptional regulation in blood stage parasites and further corroborating the lack of obvious phenotypes associated with PfAP2-HC depletion.

### The AP2 domain of PfAP2-HC is dispensable for targeting PfAP2-HC to heterochromatin

**Figure 4:**
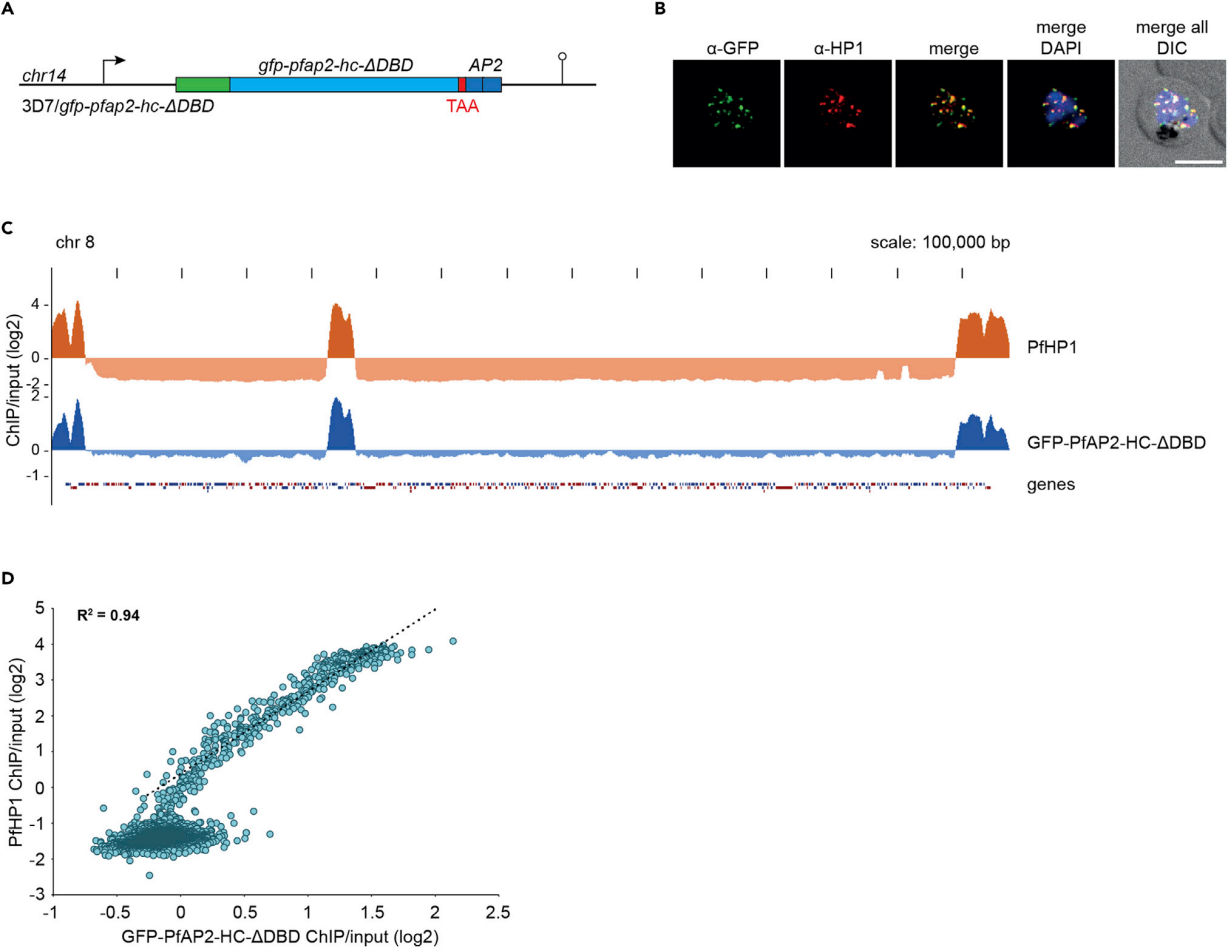
The AP2 domain of PfAP2-HC is not required for heterochromatin targeting (A) Schematic map of the *gfp-pfap2-hc* locus after CRISPR-Cas9-mediated gene editing to introduce a STOP codon prior to the sequence encoding the AP2 DBD in 3D7/GFP-PfAP2-HC-ΔDBD parasites. See also Figure S5. (B) Representative IFA images of GFP-PfAP2-HC-ΔDBD and PfHP1 localization in a developing schizont (36–44 hpi). Nuclei were stained with DAPI. DIC, differential interference contrast. Scale bar, 5 μm. (C) Log2-transformed α-PfHP1 (orange) and α-GFP (blue) ChIP-over-input tracks from 3D7/GFP-PfAP2-HC-ΔDBD schizont stage parasites. (D) Scatterplot of average log2-transformed α-PfHP1 and α-GFP ChIP-over-input values at all coding regions. The regression line is based on heterochromatic genes only (log2 ratio α-PfHP1/input ≥0). The coefficient of determination (R^2^) is displayed in the upper left corner. See also Data S1.

To discern the importance of the single AP2 DBD in targeting PfAP2-HC to heterochromatin we introduced a STOP codon prior to the AP2 domain, replacing amino acid R1319 with a premature STOP codon in 3D7/GFP-PfAP2-HC to create the parasite line 3D7/GFP-PfAP2-HC-ΔDBD (Figures 4A and S5). PCR on gDNA confirmed successful editing of the locus (Figure S5). The transgenic population consisted of a mixture of parasites either with correctly edited locus or carrying integrated donor plasmid concatemers (Figure S5). Of importance, both recombination events introduce the desired premature STOP codon into the *pfap2-hc* coding sequence. Indeed, Sanger sequencing of the amplified PCR products verified successful introduction of the premature STOP codon in the entire population (Figure S5). The localization of GFP-PfAP2-HC-ΔDBD is comparable with that of GFP-PfAP2-HC by IFA and similarly shares this localization pattern with PfHP1 (Figure 4B).

For a more comprehensive analysis, we again performed ChIP-seq experiments using α-GFP and α-PfHP1 antibodies on 3D7/GFP-PfAP2-HC-ΔDBD parasites. As with full-length GFP-PfAP2-HC, the truncated PfAP2-HC-ΔDBD protein co-localized with PfHP1 throughout the genome with highly correlated enrichment on all heterochromatic genes (Figures 4C and 4D and Data S1) showing that the AP2 DBD of PfAP2-HC is dispensable for its localization to heterochromatin.

### Binding of PfAP2-HC to heterochromatin is PfHP1 dependent

**Figure 5:**
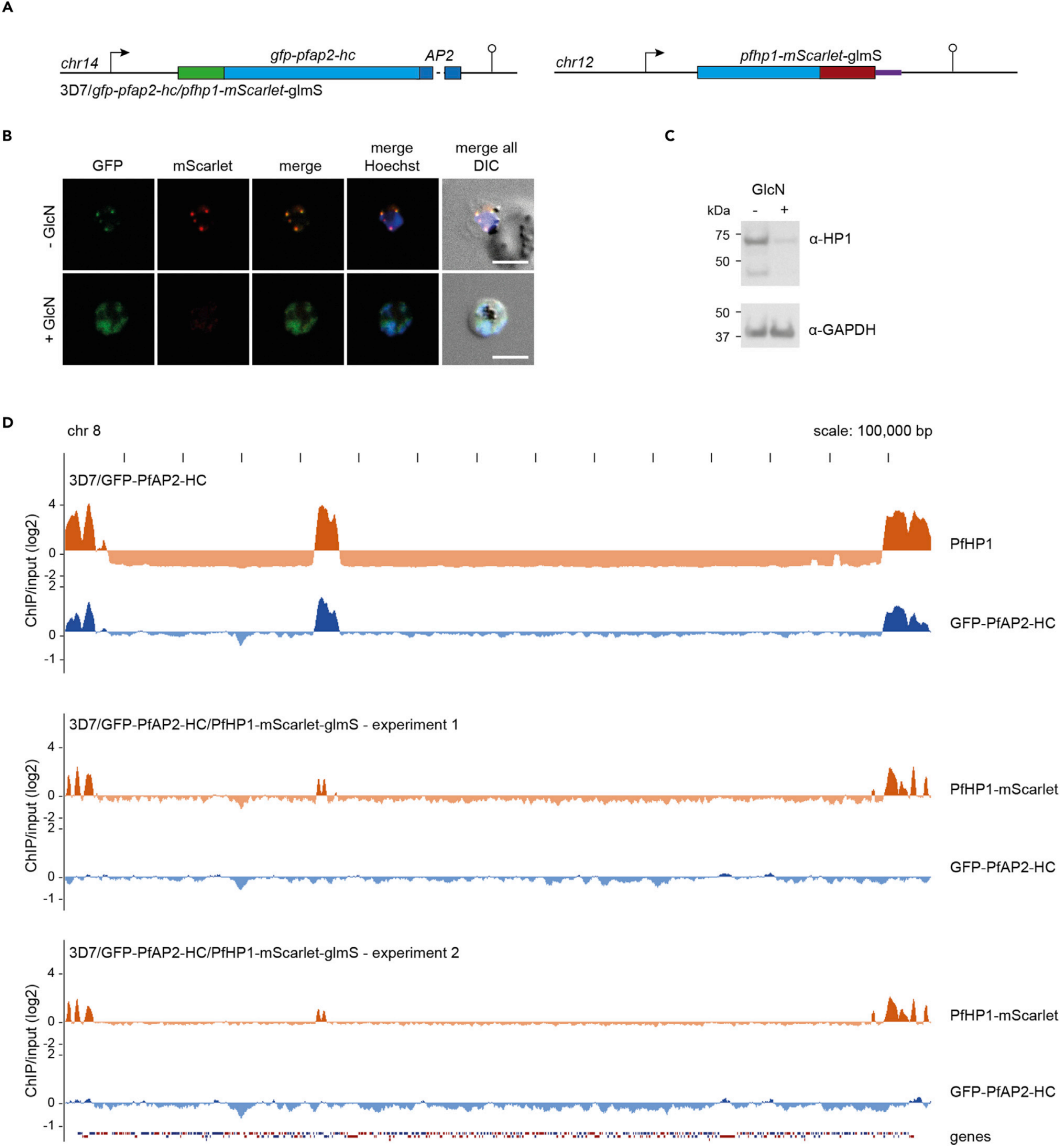
Binding of PfAP2-HC to heterochromatin is PfHP1 dependent (A) Schematic maps of the endogenous *pfap2-hc* and *pfhp1* loci after CRISPR-Cas9-based editing in 3D7/GFP-PfAP2-HC/PfHP1-mScarlet-glmS parasites. The *pfap2-hc* gene was tagged with *gfp*. The *pfhp1* gene was tagged with the *m*s*carlet* sequence followed by a *glmS* ribozyme element to allow for detection and conditional expression of PfHP1-mScarlet, respectively. See also Figure S6. (B) Representative live cell fluorescence images of 3D7/GFP-PfAP2-HC/PfHP1-mScarlet-glmS parasites at 32–40 hpi grown in the absence of GlcN (PfHP1 expressed) or the presence of GlcN (PfHP1 depleted). Nuclei were stained with Hoechst. DIC, differential interference contrast. Scale bar, 5 μm. (C) Western blot showing PfHP1-mScarlet expression levels in 3D7/GFP-PfAP2-HC/PfHP1-mScarlet-glmS schizonts (36–44 hpi) grown in the absence (−) or presence (+) of GlcN. GAPDH expression levels served as a loading control. The full-sized blot is available in Figure S6. (D) Log2-transformed α-PfHP1 (orange) and α-GFP (blue) ChIP-over-input tracks from 3D7/GFP-PfAP2-HC schizonts (top, identical to the tracks shown in Figure 1E) and from 3D7/GFP-PfAP2-HC/PfHP1-mScarlet-glmS schizonts in two independent experiments (middle and bottom).

PfAP2-HC is targeted to heterochromatin in the absence of its only recognizable DBD, suggesting a reliance on protein-protein interactions independent of the AP2 domain. To gain insight into this interaction, we tagged PfHP1 with the fluorescent protein mScarlet. In addition, we introduced a sequence encoding the *glms* riboswitch element (Prommana et al., 2013) downstream of the STOP codon, such that the resulting *pfhp1-mscarlet* mRNA contains a functional *glms* ribozyme in its 3′ untranslated region. Upon addition of glucosamine (GlcN) to the culture medium, the *glms* ribozyme mediates mRNA cleavage and degradation (Prommana et al., 2013; Watson and Fedor, 2011). We generated this conditional PfHP1 knockdown cassette in the background of the 3D7/GFP-PfAP2-HC clone to create the 3D7/GFP-PfAP2-HC/PfHP1-mScarlet-glmS double transgenic parasite line (Figures 5A and S6). We confirmed correct editing of the *pfhp1* locus by PCR on gDNA (Figure S6). To investigate the effect of PfHP1 depletion on the localization of GFP-PfAP2-HC, we split 3D7/GFP-PfAP2-HC/PfHP1-mScarlet-glmS parasites at 0–8 hpi into two populations, adding GlcN to one of them to induce the knockdown of PfHP1-mScarlet expression (+GlcN) and keeping the other one under stabilizing conditions (-GlcN). Live cell fluorescence imaging and Western blot analysis of schizont stage parasites confirmed the efficient depletion of PfHP1-mScarlet expression in +GlcN conditions (Figures 5B, 5C, and S6). Of interest, upon PfHP1-mScarlet depletion, GFP-PfAP2-HC localized diffusely throughout the nucleoplasm and no longer displayed a punctate perinuclear pattern (Figure 5B), showing mis-localization in the absence of PfHP1.

The ChIP-seq results presented in Figure 1 provided no evidence for direct binding of PfAP2-HC to DNA in euchromatic regions. However, this experiment did not allow us to test if PfAP2-HC binds to DNA sequences in heterochromatic regions because its association with PfHP1 would have masked such interactions. Hence, we used the 3D7/GFP-PfAP2-HC/PfHP1-mScarlet-glmS line to ask whether PfAP2-HC binds directly to DNA in the absence of PfHP1. We grew 3D7/GFP-PfAP2-HC/PfHP1-mScarlet-glmS parasites in the presence of GlcN from early ring stages (0–8 hpi) and harvested samples for ChIP-seq at 40–48 hpi within the same cycle. As expected, we observed a large reduction in PfHP1 enrichment in heterochromatic domains (Figure 5D). GFP-PfAP2-HC occupancy was massively reduced, and in two biologically independent ChIP-seq experiments we could not detect signals over background (Figure 5D and Data S1). Together, these results show that PfAP2-HC localization to heterochromatin is entirely dependent on PfHP1 and no evidence for direct binding of PfAP2-HC to DNA in these regions could be discerned.

### PfAP2-HC is likely not involved in heterochromatin formation

**Figure 6:**
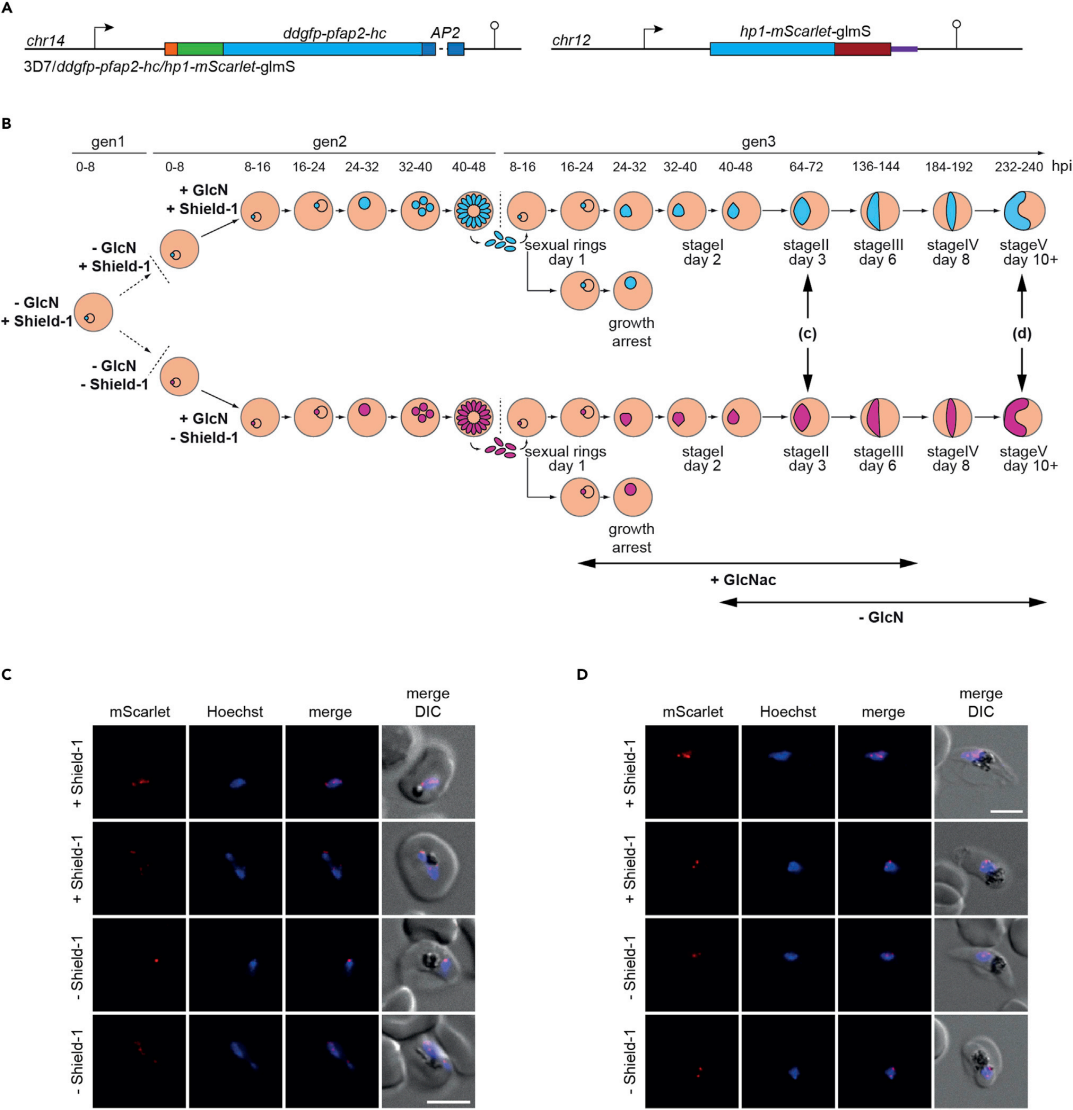
Depletion of PfAP2-HC has no marked effect on re-establishment of heterochromatin (A) Schematic map of the endogenous *pfap2-hc* and *pfhp1* loci in 3D7/DDGFP-PfAP2-HC/PfHP1-mScarlet-glmS parasites after CRISPR-Cas9-mediated gene editing. The *pfap2-hc* locus was modified to introduce a *ddgfp* tag. The *pfhp1* locus was modified to contain an *mscarlet* tag followed by the *glmS* ribozyme element. See also Figure S6. (B) Schematic detailing the design of a combined conditional DDGFP-AP2-HC depletion and PfHP1-mScarlet depletion/rescue experiment. Parasites grown in the presence of Shield-1 (+Shield-1) and the absence of glucosamine (−GlcN) exhibit stable expression of both DDGFP-PfAP2-HC and PfHP1-mScarlet. In generation 1, parasites were split into two populations at 0–8 hpi, with Shield-1 removed from one population to induce DDGFP-PfAP2-HC depletion (−Shield-1, magenta parasites) and one population maintained in the presence of Shield-1 (+Shield-1, turquoise parasites). GlcN was added to both populations at 0–8 hpi in generation 2 (+GlcN) to induce PfHP1-mScarlet depletion, which triggers sexual commitment (Brancucci et al., 2014). In generation 3, 50 mM GlcNAc was added to the ring stage cultures for 6 days to prevent growth of asexual parasites (depicted with a horizontal arrow) (Fivelman et al., 2007). Furthermore, GlcN was removed from both populations 1 day after invasion (i.e., day 2 of gametocytogenesis; stage I gametocytes) (−GlcN, horizontal arrow) to restore PfHP1-mScarlet expression during gametocytogenesis. The double vertical arrows indicate the time points of live cell fluorescence imaging experiments to assess PfHP1-mScarlet localization in DDGFP-PfAP2-HC-expressing (+Shield-1) and -depleted (−Shield-1) parasites. (C and D) Representative live cell fluorescence images showing PfHP1-mScarlet localization in stage II gametocytes (C) and stage V gametocytes (D) grown under DDGFP-PfAP2-HC-stabilizing (+Shield-1, upper two panels) and -depleting (−Shield-1, lower two panels) conditions. Nuclei were stained with Hoechst. DIC, differential interference contrast. Scale bar, 5 μm.

We have shown that maintenance and inheritance of heterochromatin was unaffected in both the 3D7/PfAP2-HC-KO null mutant and in the conditional 3D7/DDGFP-AP2-HC loss-of-function mutants after 13 generations of growth under PfAP2-HC-depleted conditions (Figure 2D). However, factors influencing the initial establishment of heterochromatin can be independent of maintenance and inheritance (Sadaie et al., 2004). Taking advantage of the fact that conditional knockdown of PfHP1 expression produces progeny consisting of approximately 50% viable heterochromatin-depleted early-stage gametocytes and 50% growth-arrested trophozoites (Brancucci et al., 2014), we investigated whether PfAP2-HC is required for the re-establishment of heterochromatin during gametocyte maturation. To achieve this, we generated a parasite line allowing for the conditional knockdown of both PfHP1 and PfAP2-HC, 3D7/DDGFP-PfAP2-HC/PfHP1-mScarlet-glmS (Figures 6A and S6). The 3D7/DDGFP-PfAP2-HC/PfHP1-mScarlet-glmS line was obtained by tagging the *pfhp1* gene in the 3D7/DDGFP-AP2-HC clone with *m*s*carlet-glmS* as described above (Figure S6). We confirmed correct editing of the *pfhp1* locus by PCR on gDNA (Figure S6). Routine culture of this parasite line in the presence of Shield-1 and absence of GlcN stabilizes DDGFP-PfAP2-HC and PfHP1-mScarlet expression, respectively. We divided ring stage parasites into two populations at 0–8 hpi (generation 1), of which one was maintained under stabilizing conditions for both proteins and from the other one Shield-1 was removed to induce DDGFP-PfAP2-HC depletion. At 0–8 hpi in generation 2, we induced the knockdown of PfHP1-mScarlet expression in both populations through addition of GlcN (Figure 6B). Both populations (DDGFP-AP2-HC stabilized/PfHP1 depleted and DDGFP-AP2-HC depleted/PfHP1 depleted) progressed into generation 3 to produce heterochromatin-depleted sexually committed parasites and growth-arrested trophozoites. On day two of gametocytogenesis, we rescued PfHP1 expression by removal of GlcN from both parasite populations and added 50 mM N-acetyl glucosamine (GlcNac) to prevent multiplication of asexual parasites (Fivelman et al., 2007; Ponnudurai et al., 1986) that can potentially arise from arrested trophozoites resuming growth after PfHP1 rescue (Brancucci et al., 2014). We then assessed the re-establishment of perinuclear heterochromatin in the presence (+Shield-1) or absence (–Shield-1) of DDGFP-AP2-HC in stage II (64–72 hpi, day 3) (Figure 6C) and stage V (232-240 hpi, day 10) (Figure 6D) gametocytes by live cell fluorescence imaging of PfHP1-mScarlet signals. We observed no marked difference in the localization pattern of PfHP1-mScarlet between gametocytes that express or do not express DDGFP-PfAP2-HC (Figures 6C and 6D). These observations indicate that PfAP2-HC likely plays no major role in *de novo* heterochromatin formation.

## Discussion

Clonally variant gene expression is key to the survival of *P. falciparum* in the human host and is dependent on heterochromatin-mediated gene silencing. PfHP1, as a core component of heterochromatin, is essential for regulating processes as diverse as antigenic variation, invasion pathway switching, commitment to gametocytogenesis, and asexual proliferation (Brancucci et al., 2014; Voss et al., 2014). Our study characterizes PfAP2-HC, a member of the ApiAP2 family of putative DNA-binding proteins that specifically associates with heterochromatin throughout the genome.

Despite progress toward understanding the heterochromatic landscape of *P. falciparum*, a global view of the dynamic processes occurring to regulate and maintain heterochromatin in this parasite remains elusive. Here, we describe PfAP2-HC as an integral component of heterochromatin, only the second such factor to be characterized after gametocyte development 1 (GDV1) (Filarsky et al., 2018). GDV1 is not expressed in asexual parasites but only in parasites undergoing sexual commitment. In these cells, GDV1 binds to heterochromatin throughout the genome and destabilizes heterochromatin particularly at the *pfap2-g* locus and early gametocyte markers thus facilitating their expression (Filarsky et al., 2018). In contrast, PfAP2-HC is expressed and binds to heterochromatin in asexual parasites. Depletion of PfAP2-HC had no effect on PfHP1 localization suggesting it is not required for heterochromatin maintenance. Factors shown to date to be involved in heterochromatin maintenance in *P. falciparum* consist of histone-modifying enzymes, such as the histone deacetylase PfHda2, whose absence leads to the expression of many PfHP1-associated genes including subtelomeric multi-gene families and the internally located *pfap2-g* locus (Coleman et al., 2014). The histone deacetylases Sir2A and Sir2B are also required for maintaining *var* gene silencing but do not appear to have a role in regulating *pfap2-g* (Duraisingh et al., 2005; Tonkin et al., 2009). The incorporation of PfHP1 into heterochromatin relies on the presence of the histone post-translational modification H3K9me3 (Kwon and Workman, 2008), which is thought to be performed by the histone lysine methyltransferase (HKMT) PfSET3 in *P. falciparum* (Cui et al., 2008). PfSET3 was identified by phylogenetic analysis as a putative ortholog of the SU(VAR)3-9 HKMTs that deposit H3K9me3 marks in model eukaryotes (Cui et al., 2008). PfSET3 was indeed localized to the nuclear periphery in *P. falciparum* (Lopez-Rubio et al., 2009; Volz et al., 2010), but so far PfSET3 has not been analyzed on the functional level and methylation of H3K9 by PfSET3 could not be proven with recombinant protein assays (Cui et al., 2008). In addition to histone-modifying enzymes, other putative PfHP1-interacting factors have been identified (Filarsky et al., 2018), although their role in heterochromatin maintenance is yet to be determined. These include the chromodomain-helicase-DNA-binding protein 1 (PfCHD1), whose homologs are important in chromatin remodeling (Bugga et al., 2013; Gaspar-Maia et al., 2009); both subunits of the FACT histone chaperone, one of which was shown as vital in the production of fertile male gametes in *P. berghei* (Laurentino et al., 2011); and the Pf14-3-3I reader protein that specifically recognizes phosphorylation of serine 28 on histone 3 (Dastidar et al., 2013). The manner in which all these chromatin components interact and cooperate to mediate reversible gene silencing in *P. falciparum* is an interesting and equally challenging question for future research.

We also tested whether the absence of PfAP2-HC may influence heterochromatin formation rather than maintenance. Because PfHP1 is essential for the proliferation of asexual parasites, we performed this experiment in gametocytes where PfHP1 is dispensable (Brancucci et al., 2014). To this end, we first depleted PfHP1 in 3D7/DDGFP-PfAP2-HC/PfHP1-mScarlet-glmS parasites through conditional knockdown of PfHP1 expression and then rescued PfHP1 expression in the sexual ring stage progeny and visualized the re-establishment of heterochromatic foci in stage II and V gametocytes by fluorescence microscopy based on PfHP1-mScarlet positivity. We did not observe any difference in the localization of PfHP1 between gametocytes expressing or not expressing PfAP2-HC. This result provides preliminary evidence suggesting that *de novo* formation of heterochromatin occurs independent of PfAP2-HC. This is in keeping with our observation that PfAP2-HC does not seem to bind chromosomal DNA *in vivo* and that the localization of PfAP2-HC is dependent on the presence of PfHP1, as discussed further below. However, we cannot exclude the possibility that a role for PfAP2-HC in nucleating heterochromatin may have been masked in this experiment by the spreading of heterochromatin from residual PfHP1 foci that remained bound to chromatin owing to incomplete PfHP1 knockdown in the asexual progenitors (Figure 5D).

We showed that the AP2 DBD of PfAP2-HC is not required for correct localization of the protein to heterochromatin. Furthermore, we could not detect direct binding of PfAP2-HC to the predicted CACACA target motifs (Campbell et al., 2010) or to other sites in chromosomal DNA *in vivo* by ChIP-seq, neither in euchromatin nor in heterochromatin, and PfAP2-HC depletion had no effect on gene transcription during the IDC. In addition, recent pull-down experiments of native nuclear proteins binding to specific DNA probes also failed to reveal an interaction of full-length PfAP2-HC with the CACACA motif (Toenhake et al., 2018). Together, these results imply that PfAP2-HC does not bind chromosomal DNA *in vivo*, suggesting functional divergence of AP2 domains within the ApiAP2 family. Although DNA-binding motifs were identified for most AP2 domains *in vitro* (Campbell et al., 2010), two of the three AP2 domains of PfAP2-I were recently shown to be dispensable in the IDC and it is unknown if they actually bind DNA *in vivo* (Santos et al., 2017). It is still possible that any direct DNA binding of PfAP2-HC was below the detection limit of our ChIP-seq experiments. However, it is perhaps more likely that PfAP2-HC indeed does not bind DNA directly *in vivo*, given its dependence on PfHP1 for correct localization. In fact, because PfAP2-HC interacts with heterochromatin independent of its AP2 domain, PfAP2-HC may actually not be meant to bind DNA directly; PfAP2-HC would likely recruit heterochromatin to any chromosomal sites it would bind to and thus potentially silence expression of genes that are important for parasite viability.

The apparent lack of DNA-binding activity displayed by the PfAP2-HC AP2 domain and the capacity of PfAP2-HC to localize to heterochromatin in absence of the AP2 domain suggest that protein-protein interactions involving the large N terminus of the protein are responsible for targeting PfAP2-HC to heterochromatin. Multiple sequence alignments of AP2-HC orthologs across all human-infecting *Plasmodium* spp. show only 30%–36% sequence identity to PfAP2-HC, and this is comparable with the AP2-HC orthologs of rodent-infecting species (31%–32%) (Figure S7). High sequence similarity is mainly confined to the AP2 domain itself, which shares ≥90% identical amino acids across all species (Figure S7). Of interest, there is a second semi-conserved region of 172 amino acids within PfAP2-HC with 64%–67% sequence identity to the orthologs of other human-infecting species and 53%–56% identity to those from rodent-infecting species (Figure S7), which points to an evolutionarily conserved feature. One could speculate that this region may be involved in mediating interactions with PfHP1 or other chromatin-associated factors. To date, the role of the non-AP2 region of ApiAP2 proteins has not been explicitly studied. However, given the regulatory roles PfAP2-G (Josling et al., 2020; Kafsack et al., 2014), PfAP2-I (Santos et al., 2017), and PfAP2-EXP (Martins et al., 2017) play as transcription factors, as well as the *P. berghei* ApiAP2 factors PbAP2-G (Sinha et al., 2014), PbAP2-G2 (Yuda et al., 2015), PbAP2-FG (Yuda et al., 2020), PbAP2-O (Kaneko et al., 2015; Yuda et al., 2009), PbAP2-Sp (Yuda et al., 2010), and PbAP2-L (Iwanaga et al., 2012), it can be assumed that these regions are involved in recruiting transcriptional and epigenetic machinery to the promoters in question. Indeed, coIP experiments identified the bromodomain protein PfBDP1, PfCHD1, and the FACT complex as potential interaction partners of PfAP2-I (Santos et al., 2017) and truncation of PfAP2-EXP to express only the AP2 domain led to de-regulation of its target genes (Martins et al., 2017). Functional analysis of the semi-conserved region identified in PfAP2-HC may be a promising starting point to begin understanding the role of non-AP2 domain regions in ApiAP2 factor function.

The AP2-HC factor is conserved among all *Plasmodium* spp., which clearly suggests an important role for this factor in the biology of malaria parasites, at least *in vivo*. We obtained a viable PfAP2-HC KO line that lacks any obvious phenotype in asexual blood stage parasites, but we cannot rule out functionally critical roles in other life cycle stages. Indeed, RNA-seq data show *pfap2-hc* expression in gametocyte and sporozoite stages (plasmodb.org) (Aurrecoechea et al., 2009; Gomez-Diaz et al., 2017; Lasonder et al., 2016). However, the orthologs of PfAP2-HC were successfully disrupted in the rodent malaria parasites *P. berghei* and *P. yoelii*, without discernible growth defects observed during the full life cycle in laboratory animals (Modrzynska et al., 2017; Zhang et al., 2017). These results suggest that functional redundancy or compensatory mechanisms may exist among the ApiAP2 family, as also proposed by Zhang and colleagues (Zhang et al., 2017). However, at least in asexual blood stage parasites, we believe mechanisms compensating for loss of PfAP2-HC function are highly unlikely given that the conditional knockdown of PfAP2-HC expression did not result in any transcriptional changes and caused not even a temporary defect on parasite growth or multiplication. Beyond this, it is also possible that PfAP2-HC is involved in more subtle processes not studied here, which may not present as immediate phenotypes in loss-of-function mutants but may be crucial for parasite fitness in the field. Examples of such processes are DNA repair/recombination within heterochromatic regions or epigenetic memory/switching frequencies of heterochromatic genes. The heterochromatic subtelomeric regions, which contain several hundred members of multi-copy gene families, recombine at a higher rate than the core genome in *P. falciparum*, resulting in high antigenic diversity within the parasite population (Bopp et al., 2013; Claessens et al., 2014; Frank et al., 2008). Furthermore, DNA repair mechanisms are generally less efficient in heterochromatin compared with euchromatin and thus contribute to increased mutation rates in these regions (Fortuny and Polo, 2018; Mao and Wyrick, 2019). Switches in the transcription of heterochromatic genes creates clonal variation in the expression of surface antigens, invasion factors, nutrient channels, or PfAP2-G, allowing the parasite population to adapt to and survive under adverse environmental conditions (Cortes and Deitsch, 2017; Llora-Batlle et al., 2020; Voss et al., 2014). Activation of silenced heterochromatic genes is linked to local chromatin remodeling, as demonstrated for *var* genes (Brancucci et al., 2014; Chookajorn et al., 2007; Lopez-Rubio et al., 2007), *pfap2-g* (Brancucci et al., 2014; Filarsky et al., 2018), and other clonally variant genes (Crowley et al., 2011). As an integral and specific component of heterochromatin, it is at least conceivable that PfAP2-HC may act as a positive or negative regulator of DNA repair or chromatin remodeling processes in heterochromatic regions.

In summary, our study provides a comprehensive analysis of the ApiAP2 factor PfAP2-HC, based on the analysis of six different single or double engineered transgenic parasite lines. Along with PfAP2-Tel (Sierra-Miranda et al., 2017) and PfSIP2 (Flueck et al., 2010), PfAP2-HC joins the ranks of ApiAP2 factors that do not primarily act as transcriptional regulators. We rather characterized PfAP2-HC as a PfHP1-interacting protein and core component of heterochromatin in *P. falciparum*. We found no evidence for direct binding of PfAP2-HC to chromosomal DNA *in vivo* and show that the localization of PfAP2-HC to heterochromatin is independent of the AP2 domain but strictly dependent on the presence of PfHP1. Although our efforts failed to reveal conclusive insight into PfAP2-HC function, we discovered unexpected properties of ApiAP2 factors that highlight the functional diversity among the members of this family of putative DNA-binding proteins.

### Limitations of the study

As we did not observe any PfAP2-HC loss-of-function phenotypes in *P. falciparum* blood stage parasites in our study, targeted experiments in other life cycle stages will be necessary to reveal insight into the function of this ApiAP2 factor. Furthermore, although our preliminary microscopy-based data presented in Figure 6 suggest that PfAP2-HC is not involved in *de novo* heterochromatin formation, ChIP-seq and RNA-seq experiments would be required to confirm this result at higher resolution.

### Resource availability

#### Lead contact

Further information and requests for resources and reagents should be directed to and will be fulfilled by the Lead Contact, Till Steffen Voss (till.voss@swisstph.ch).

#### Materials availability

Parasite lines and plasmid constructs are available from the authors upon request.

#### Data and code availability

The ChIP-seq and microarray data reported in this publication have been deposited in NCBI's Gene Expression Omnibus (Edgar et al., 2002) and are accessible through GEO Series accession numbers GSE154840 and GSE159061, respectively. Additional data that support the findings of this study are available in Data S1 and S2.

## Methods

All methods can be found in the accompanying Transparent methods supplemental file.

## Supplemental information

Document S1. Transparent methods, Figures S1–S7, and Tables S1 and S2

Data S1. ChIP-seq enrichment values, related to figures 1, 2, and 4 ChIP/input enrichment values were calculated over each coding region of the *P. falciparum* 3D7 reference genome from PlasmoDB v26 (www.plasmodb.org) in 3D7/GFP-PfAP2-HC, 3D7/DDGFP-PfAP2-HC ON and OFF Shield-1, 3D7/WT, 3D7/PfAP2-HC-KO, 3D7/GFP-PfAP2-HC-ΔDBD, and 3D7/GFP-PfAP2-HC/PfHP1-mScarlet-glmS parasites at 36–44 hpi. Columns A-B: Gene ID and gene annotation. Column C: 3D7/GFP-PfAP2-HC GFP ChIP/input values. Column D: 3D7/GFP-PfAP2-HC HP1 ChIP/input values. Column E: 3D7/DDGFP-PfAP2-HC ON Shield-1 HP1 ChIP/input values. Column F: 3D7/DDGFP-PfAP2-HC OFF Shield-1 HP1 ChIP/input values. Column G: 3D7/WT HP1 ChIP/input values. Column H: 3D7/PfAP2-HC-KO HP1 ChIP/input values. Column I: 3D7/GFP-PfAP2-HC-ΔDBD GFP ChIP/input values. Column J: 3D7/GFP-PfAP2-HC-ΔDBD HP1 ChIP/input values. Column K: 3D7/GFP-PfAP2-HC/PfHP1-mScarlet-glmS GFP ChIP/input values (Experiment 1). Column L: 3D7/GFP-PfAP2-HC/PfHP1-mScarlet-glmS HP1 ChIP/input values (Experiment 1). Column M: 3D7/GFP-PfAP2-HC/PfHP1-mScarlet-glmS GFP ChIP/input values (Experiment 2). Column N: 3D7/GFP-PfAP2-HC/PfHP1-mScarlet-glmS HP1 ChIP/input values (Experiment 2).

Data S2. Processed microarray data obtained from 3D7/DDGFP-PfAP2-HC parasites, related to figure 3 Columns A-B: Gene ID and gene annotation. Column C: HP1 target genes (Flueck et al., 2009; Fraschka et al., 2018). Columns D-H: Cy5/Cy3 log2 ratios for all transcripts and five TPs (8–16 hpi, 16–24 hpi, 24–32 hpi, 32–40 hpi, 40–48 hpi) harvested from 3D7/DDGFP-PfAP2-HC parasites cultured in presence of Shield-1 (ON). Columns I-M: Cy5/Cy3 log2 ratios for all transcripts and five TPs harvested from 3D7/DDGFP-PfAP2-HC parasites cultured in absence of Shield-1 (OFF). Columns N-R: fold change in gene expression (log2) in 3D7/DDGFP-PfAP2-HC parasites cultured in absence Shield-1 (OFF) compared with 3D7/DDGFP-PfAP2-HC parasites cultured in presence of Shield-1 (ON) for each of the five paired TPs. Column S: mean fold change in gene expression across all five TPs. Column T: p-value (paired two-tailed Student's t-test). Column U: negative log10 of the p-value.
