# Supplementary material for: The ApiAP2 factor PfAP2-HC is an integral component of heterochromatin in the malaria parasite Plasmodium falciparum
Source: iScience. 2021 Apr 16;24(5):102444. doi: 10.1016/j.isci.2021.102444 (PMC8105651; doi:10.1016/j.isci.2021.102444)
Supplement: Document S1. Transparent methods, Figures S1–S7, and Tables S1 and S2 [file mmc1.pdf]

iScience, Volume *24*

Supplemental information

The ApiAP2 factor PfAP2-HC is an integral component of heterochromatin in the malaria parasite *Plasmodium falciparum*

Eilidh Carrington, Roel Henrikus Martinus Cooijmans, Dominique Keller, Christa Geeke Toenhake, Richárd Bártfai, and Till Steffen Voss

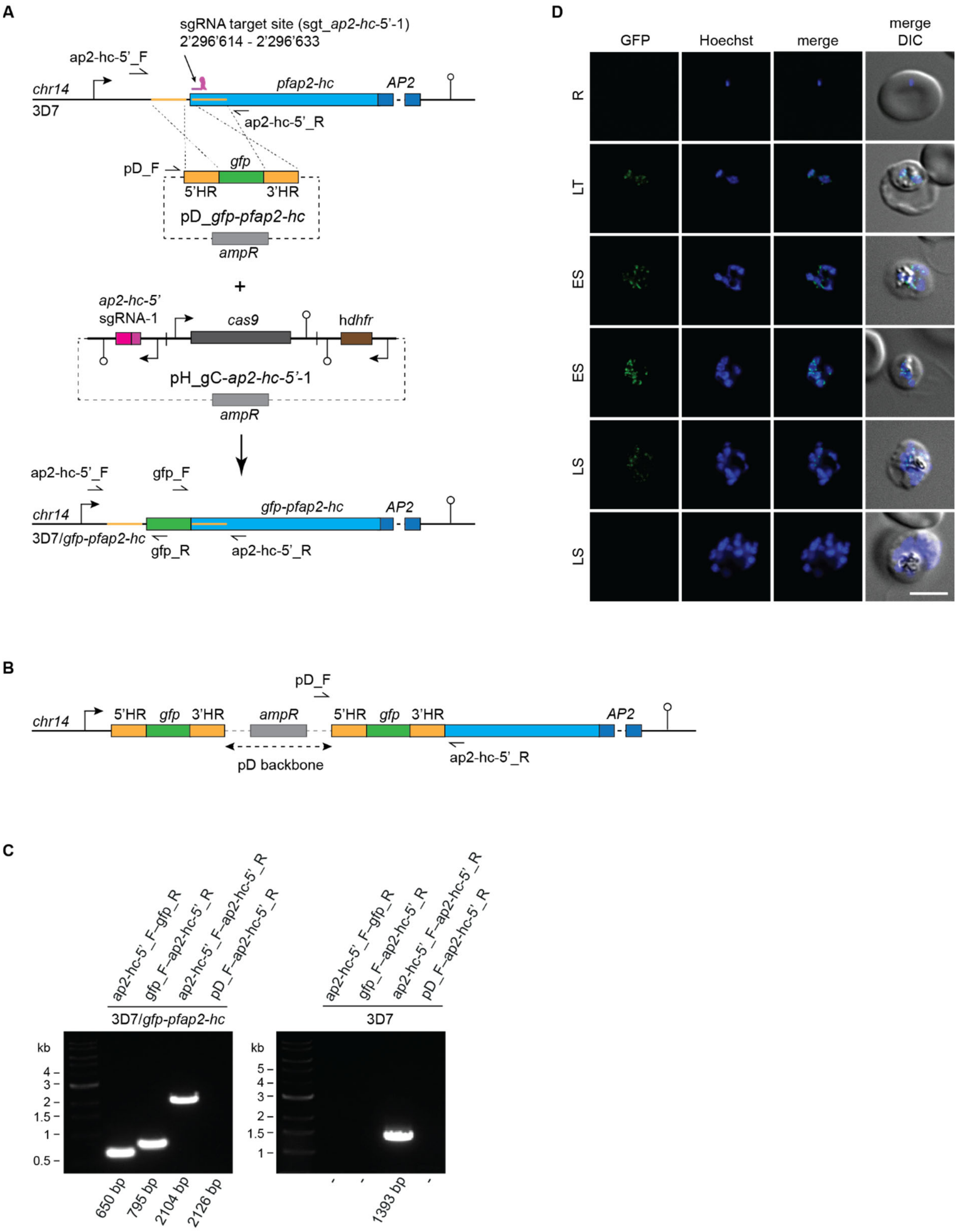

Figure S1. Generation of the 3D7/GFP-PfAP2-HC parasite line and live cell fluorescence imaging, Related to Figure 1

(A) Schematic maps of the *pfap2-hc* locus (PF3D7_1456000) in 3D7 parasites (top), the CRISPR/Cas9 transfection vectors pD_*gfp-pfap2-hc* and pH_gC-*ap2-hc-5'*-1 (centre), and the modified *pfap2-hc* locus after CRISPR/Cas9-based genome editing in 3D7/GFP-PfAP2-HC parasites (bottom). The AP2 DBD-encoding sequence, which is interrupted by an intron, is indicated (AP2, dark blue). The position of the sgt_*ap2-hc-5'*-1 sgRNA target sequence is indicated (chromosome 14 coordinates). The pD_*gfp-pfap2-hc* donor plasmid contains a *gfp* sequence (green) flanked by homology regions (HR, yellow) for homology-directed repair. The pH_gC-*ap2-hc-5'*-1 plasmid contains expression cassettes for SpCas9 (dark grey), the sgRNA (pink) and the h*dhfr* resistance marker

(brown). Successful gene editing results in the expression of an N-terminally tagged GFP-PfAP2-HC protein. PCR primer binding sites are indicated by arrows and were used to confirm successful gene editing.
(B) Schematic map of the modified *pfap2-hc* locus after CRISPR/Cas9-based genome editing in the event of donor plasmid concatemer integration into the genome. PCR primer binding sites are indicated by arrows and were used to check for donor plasmid concatemer integration.
(C) PCR on gDNA from a 3D7/GFP-PfAP2-HC clone and 3D7 wild-type parasites. Primers ap2-hc-5'_F and ap2-hc-5'_R bind to chromosomal sequences outside the HRs and amplify a 2104 bp or 1393 bp fragment from the edited or wild-type *pfap2-hc* locus, respectively. The ap2-hc-5'_F-gfp_R and gfp_F-ap2-hc-5'_R primer combinations are specific for the edited locus and amplify 650 bp and 795 bp fragments, respectively. Primer pD_F binds to the donor plasmid backbone and, when used in combination with primer ap2-hc-5'_R, will amplify a fragment of 2126 bp if a donor plasmid concatemer was integrated into the genome.
(D) Live cell fluorescence imaging of 3D7/GFP-PfAP2-HC parasites throughout the IDC. R, ring stage. LT, late trophozoite with two parasites infecting one RBC. ES, early schizont. LS, late schizont. Nuclei were stained with Hoechst. DIC, differential interference contrast. Scale bar, 5 µm.

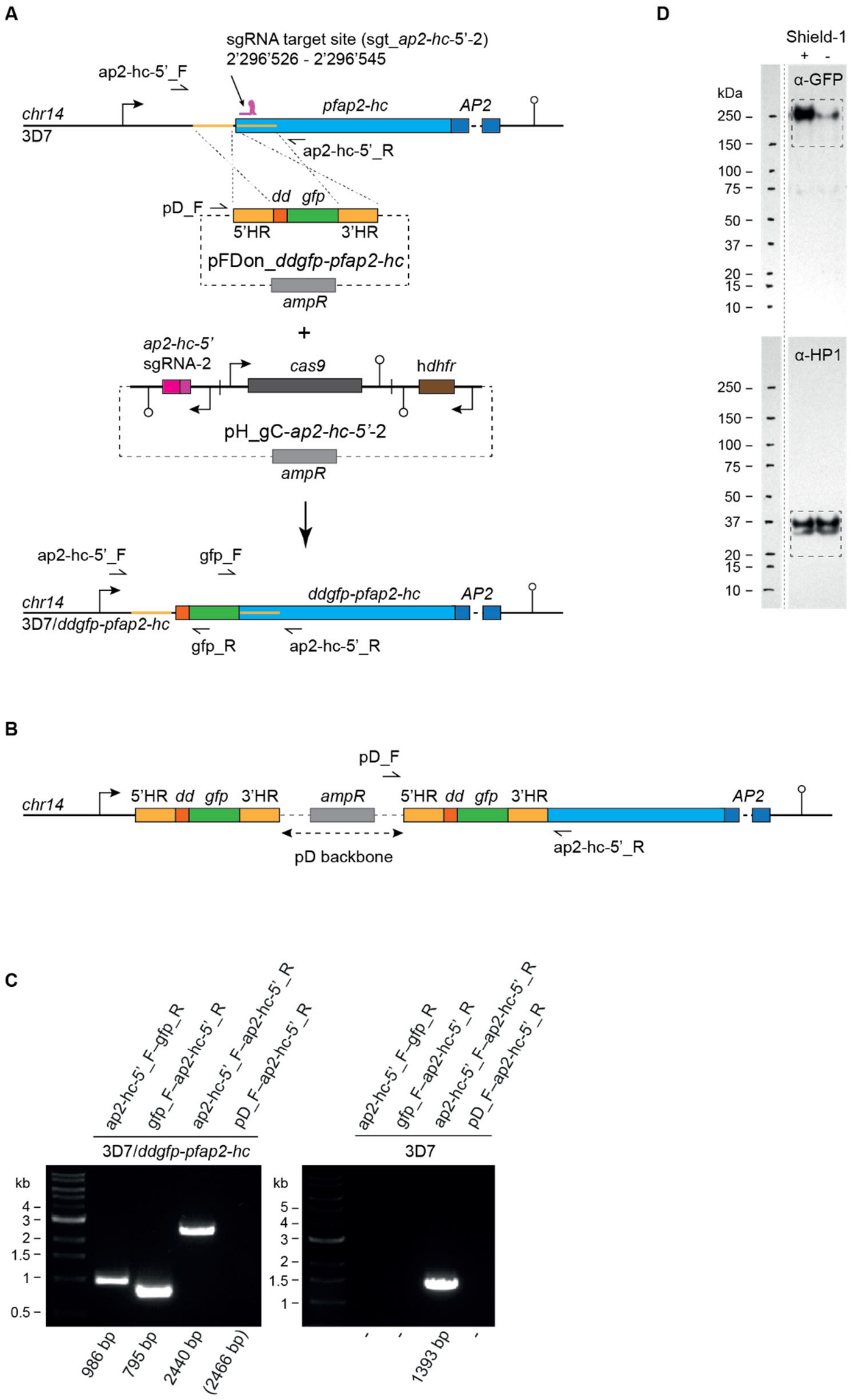

Figure S2. Generation of the 3D7/DDGFP-PfAP2-HC parasite line, Related to Figure 2
(A) Schematic maps of the *pfap2-hc* locus (PF3D7_1456000) in 3D7 parasites (top), the CRISPR/Cas9 transfection vectors pFDon_*ddgfp-pfap2-hc* and pH_gC-*ap2-hc-5'*-2 (centre), and the modified *pfap2-hc* locus after CRISPR/Cas9-based genome editing in 3D7/DDGFP-PfAP2-HC parasites (bottom). The AP2 DBD-encoding sequence, which is interrupted by an intron, is indicated (AP2, dark blue). The position of the sgt_*ap2-hc-5'*-1 sgRNA target sequence is indicated (chromosome 14 coordinates). The pFDon_*ddgfp-pfap2-hc* donor plasmid contains an FKBP destabilisation domain (*dd*, orange) and *gfp* sequence (green) flanked by homology regions (HR, yellow) for homology-directed repair. The pH_gC-*ap2-hc-5'*-2 plasmid contains expression cassettes for SpCas9 (dark grey), the sgRNA (pink) and the h*dhfr* resistance marker (brown). Successful gene

editing results in the expression of an N-terminally tagged DDGFP-PfAP2-HC protein. PCR primer binding sites are indicated by half arrows and were used to confirm successful gene editing.

(B) Schematic map of the modified *pfap2-hc* locus after CRISPR/Cas9-based genome editing in the event of donor plasmid concatemer integration into the genome. PCR primer binding sites are indicated by arrows and were used to check for donor plasmid concatemer integration.

(C) PCR on gDNA from a 3D7/DDGFP-PfAP2-HC clone and 3D7 wild-type parasites. Primers ap2-hc-5'_F and ap2-hc-5'_R bind to chromosomal sequences outside the HRs and amplify a 2440 bp or 1393 bp fragment from the edited or wild-type *pfap2-hc* locus, respectively. The ap2-hc-5'_F-gfp_R and gfp_F-ap2-hc-5'_R primer combinations are specific for the edited locus and amplify 986 bp and 795 bp fragments, respectively. Primer pD_F binds to the donor plasmid backbone and, when used in combination with primer ap2-hc-5'_R, will amplify a fragment of 2466 bp if a donor plasmid concatemer was integrated into the genome.

(D) Full sized Western blot of the sections shown in Figure 2C showing DDGFP-PfAP2-HC expression levels in 3D7/DDGFP-PfAP2-HC parasites grown in the presence (+) or absence (-) of Shield-1. The membrane was first probed with α-GFP antibodies (top) before inactivation of horseradish peroxidase with 2 mM NaN_3, followed by re-probing with the α-PfHP1 antibodies (bottom) used as a loading control. Dashed boxes show the sections presented in Figure 2C.

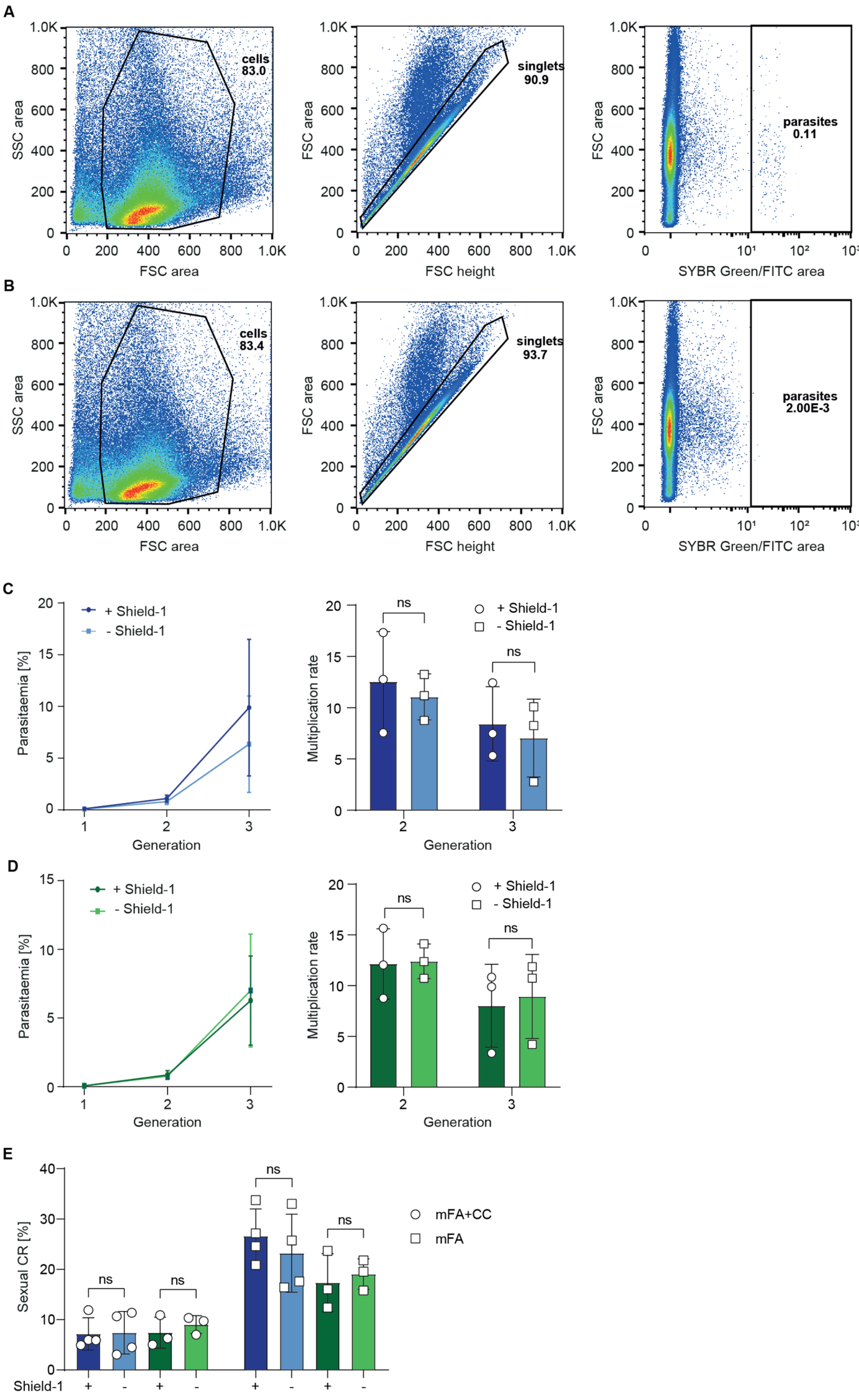

Figure S3. Multiplication rates and gametocyte conversion rates of 3D7/DDGFP-PfAP2-HC parasites, Related to Figure 2
(A, B) Gating strategy applied to flow cytometry data obtained from multiplication assays. Representative flow cytometry plots of an infected (3D7/WT, – Shield-1, panel A) and uninfected RBC control sample (panel B) measured on day 1 of the multiplication assay. The first plot shows the gate

to remove debris smaller than cell size to include only the ‘cells’ population. The second plot shows the gate to include only single measurement events, termed ‘singlets’, and the third gate separates uninfected from infected RBCs based on the SYBR Green intensity of the uninfected RBC control, termed ‘parasites’. The numbers are the percentage of events included within the gate, with the final gate ‘parasites’ reflecting the parasitaemia of the sample. This gating strategy was applied to all flow cytometry data shown in panels C and D, and in Figure S4.
(C, D) Flow cytometry data showing the increase in parasitaemia (left) and parasite multiplication rates (right) in two subsequent generations of 3D7/DDGFP-PfAP2-HC (panel C) parasites grown in the presence (+, dark blue) or absence (–, light blue) of Shield-1 and 3D7/WT (panel D) parasites grown in the presence (+, dark green) or absence (–, light green) of Shield-1. The mean ±SD of three biological replicates are shown. Data points of individual replicates are shown for parasite multiplication rates and represented by open circles (+ Shield-1) or open squares (– Shield-1). ns, not significant (paired two-tailed Student’s t test).
(E) Sexual conversion rates of 3D7/DDGFP-PfAP2-HC (dark/light blue) and 3D7/WT (dark/light green) parasites exposed to mFA+CC medium (conditions inhibiting sexual commitment, open circles) or mFA medium (conditions inducing sexual commitment, open squares) (Brancucci et al., 2017). Parasites grown in the presence (+) or absence (–) of Shield-1 are compared. The mean ±SD of four biological replicates of 3D7/DDGFP-PfAP2-HC and three biological replicates of 3D7/WT are shown. Data points of individual replicates are shown. ns, not significant (paired two-tailed Student’s t test). CR, conversion rate.

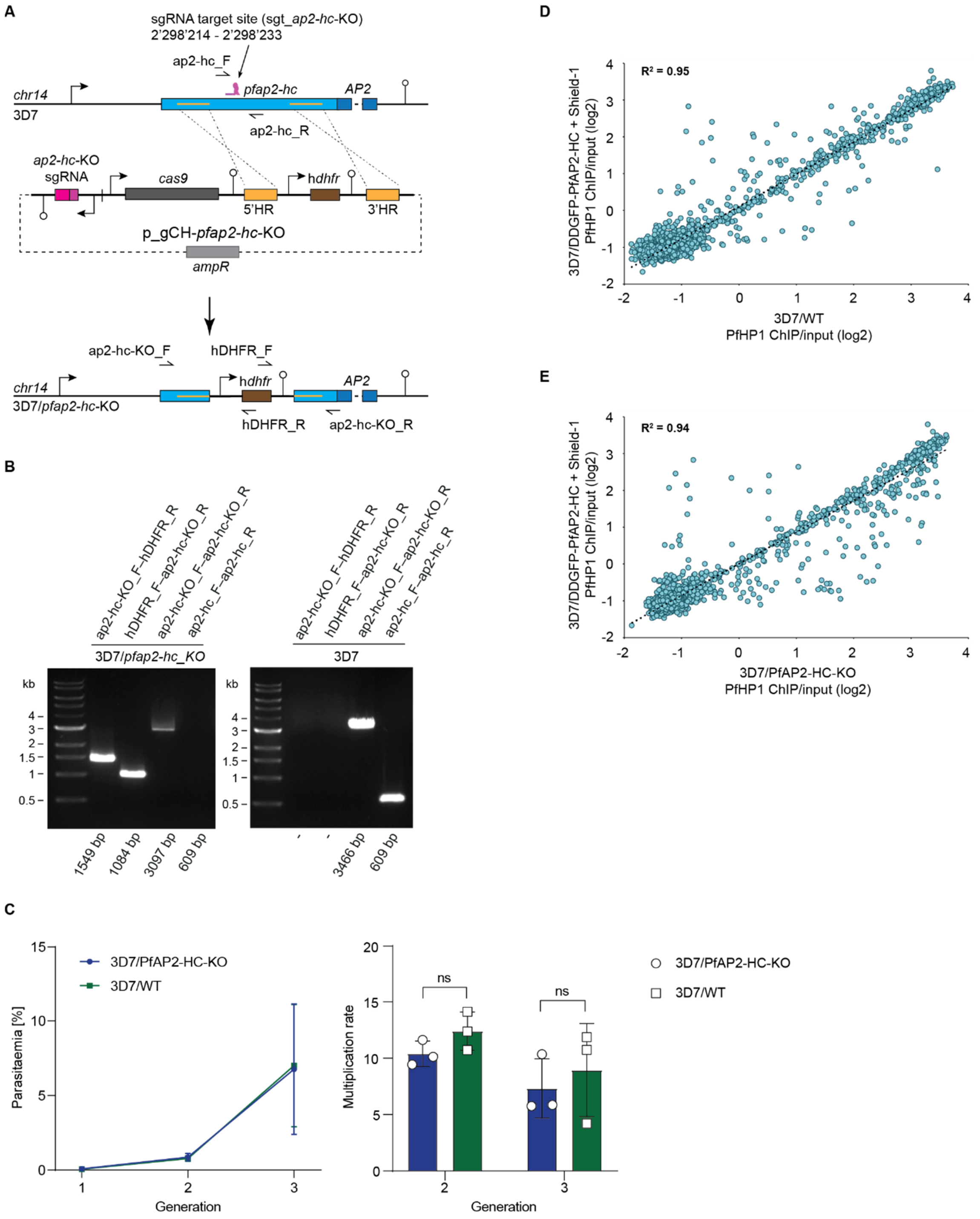

Figure S4. Generation of the 3D7/PfAP2-HC-KO parasite line, Related to Figure 2
(A) Schematic maps of the *pfap2-hc* locus (PF3D7_1456000) in 3D7 parasites (top), the p_gCH-*pfap2-hc*-KO transfection vector (centre), and the modified *pfap2-hc* locus after CRISPR/Cas9-based genome editing in 3D7/PfAP2-HC-KO parasites (bottom). The AP2 DBD-encoding sequence, which is interrupted by an intron, is indicated (AP2, dark blue). The position of the sgt_*ap2-hc*-KO sgRNA target sequence is indicated (chromosome 14 coordinates). The p_gCH-*pfap2-hc*-KO plasmid contains expression cassettes for SpCas9 (dark grey), the sgRNA (pink) and the h*dhfr* resistance marker (brown) flanked by two homology regions (HR, yellow) for homology-directed repair. Successful gene editing results in the h*dhfr* expression cassette replacing a section of the *pfap2-hc* gene, disrupting its expression. PCR primer binding sites are indicated by arrows and were used to confirm successful gene editing.

(B) PCR on gDNA from 3D7/PfAP2-HC-KO and 3D7 wild-type parasites. Primers ap2-hc-KO_F and ap2-hc-KO_R bind to chromosomal sequences outside the HRs and amplify a 3097 bp or 3466 bp fragment from the edited or wild-type *pfap2-hc* locus, respectively. The ap2-hc-KO_F-hDHFR_R and hDHFR_F-ap2-hc-KO_R primer combinations are specific for the edited locus and amplify 1549 bp and 1084 bp fragments, respectively. Primer combination ap2-hc_F-ap2-hc_R is specific for the wild-type locus and amplifies a 609 bp fragment.
(C) Flow cytometry data showing the increase in parasitaemia (left) and parasite multiplication rates (right) in two subsequent generations of 3D7/PfAP2-HC-KO (dark blue) and 3D7/WT (dark green) parasites. The 3D7/WT data is identical to those shown in Figure S2D (3D7/WT grown in the absence of Shield-1). The mean ±SD of three biological replicates are shown. Data points of individual replicates are shown for parasite multiplication rates and represented by open circles (3D7/PfAP2-HC-KO) or open squares (3D7/WT). ns, not significant (paired two-tailed Student's t test).
(D, E) Scatterplots of average log2-transformed α-PfHP1 ChIP/input values for all parasite genes in 3D7/WT and 3D7/DDGFP-PfAP2-HC schizonts grown in the presence (+) of Shield-1 (panel C) and in 3D7/PfAP2-HC-KO and 3D7/DDGFP-PfAP2-HC schizonts grown in the presence (+) of Shield-1 (panel D). Depicted regression lines are based on heterochromatic genes only (log2 ratio α-PfHP1/input ≥ 0). The coefficients of determination (R^2) are shown on the top left.

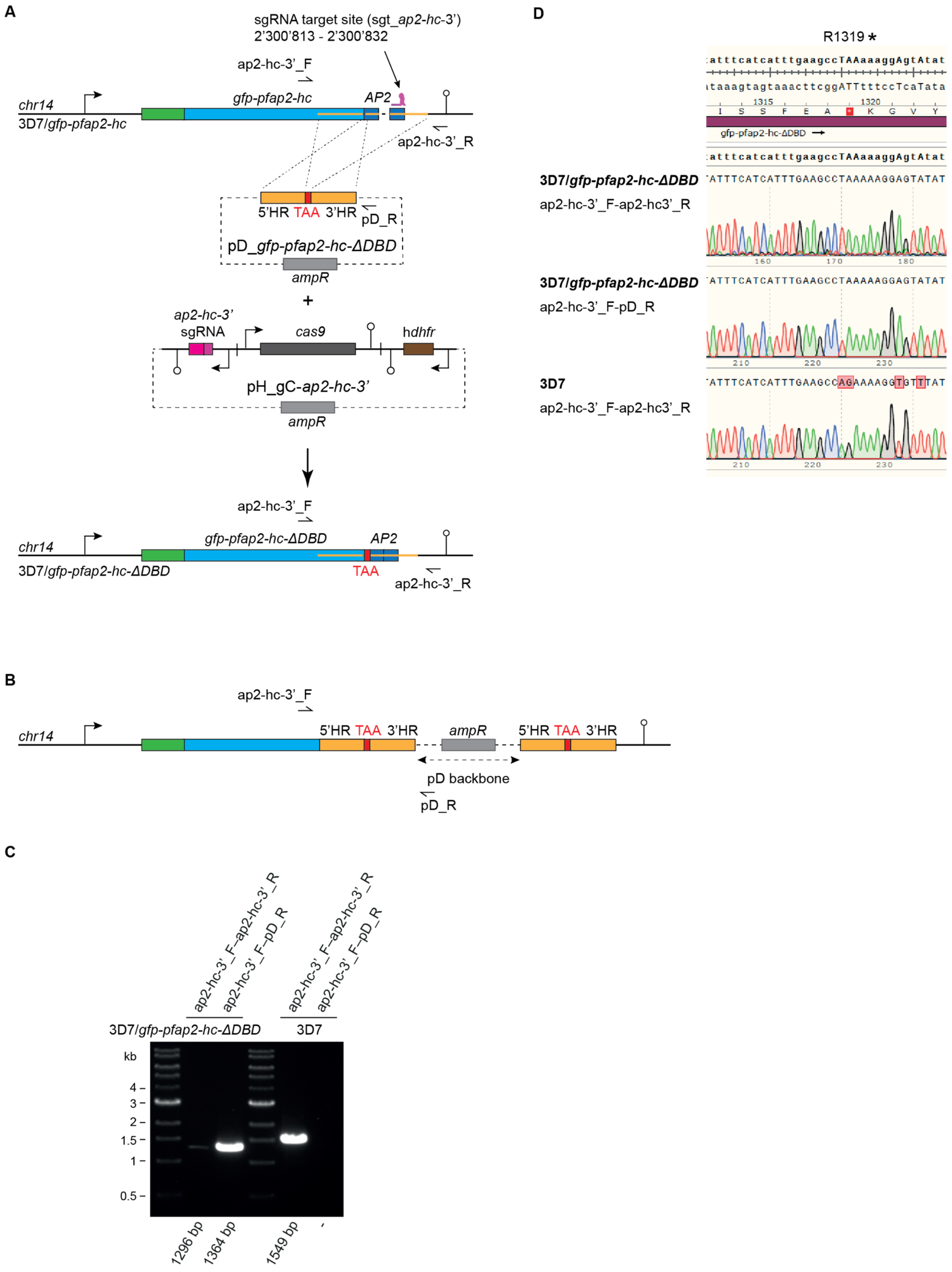

Figure S5. Generation of the 3D7/GFP-PfAP2-HC-ΔDBD parasite line, Related to Figure 4
(A) Schematic maps of the *gfp-pfap2-hc* locus in 3D7/GFP-PfAP2-HC parasites (top, see Figure S1), the CRISPR/Cas9 transfection vectors pD_*gfp-pfap2-hc-ΔDBD* and pH_gC-*ap2-hc-3'* (centre), and the modified *gfp-pfap2-hc* locus after CRISPR/Cas9-based genome editing in 3D7/GFP-PfAP2-HC-ΔDBD parasites (bottom). The AP2 DBD-encoding sequence, which is interrupted by an intron, is indicated (AP2, dark blue). The position of the sgt_*ap2-hc-3'* sgRNA target sequence is indicated (chromosome 14 coordinates). The pD_*gfp-pfap2-hc-ΔDBD* donor plasmid contains a premature TAA stop codon

(red) flanked by homology regions (HR, yellow) for homology-directed repair. The pH_gC-*ap2-hc-3'* plasmid contains expression cassettes for SpCas9 (dark grey), the sgRNA (pink) and the h*dhfr* resistance marker (brown). Successful gene editing results in the expression of a truncated GFP-PfAP2-HC protein lacking the AP2 DNA-binding domain (GFP-PfAP2-HC-ΔDBD). PCR primer binding sites are indicated by arrows and were used to confirm successful gene editing.
(B) Schematic map of the modified *gfp-pfap2-hc* locus after CRISPR/Cas9-based genome editing in the event of donor plasmid concatemer integration into the genome. PCR primer binding sites are indicated by arrows and were used to check for donor plasmid concatemer integration.
(C) PCR on gDNA from 3D7/GFP-PfAP2-HC-ΔDBD and 3D7 wild-type parasites. Primers ap2-hc-3'_F and ap2-hc-3'_R bind to chromosomal sequences outside the HRs and amplify a 1296 bp or 1549 bp fragment from the edited or wild-type *pfap2-hc* locus, respectively. Primer pD_R binds to the donor plasmid backbone and, when used in combination with primer ap2-hc-3'_F, amplifies a fragment of 1364 bp if a donor plasmid concatemer was integrated into the genome.
(D) Sanger sequencing of the two PCR products ap2-hc-3'_F-ap2-hc-3'_R (top) and ap2-hc-3'_F-pD_R (middle) amplified from 3D7/GFP-PfAP2-HC-ΔDBD parasites (see panel B, lanes 2 and 3) confirms the successful introduction of the AG→TA double mutation creating a premature STOP codon (R1319*). The PCR product ap2-hc-3'_F-ap2-hc-3'_R (bottom) amplified from 3D7 wild-type parasites (see panel B, lane 5) shows the wild-type sequence. Additional mutations downstream of the AG→TA double mutation are part of the re-codonised sequence introduced to avoid homologues recombination at an undesired location to ensure correct CRISPR/Cas9 genome editing.

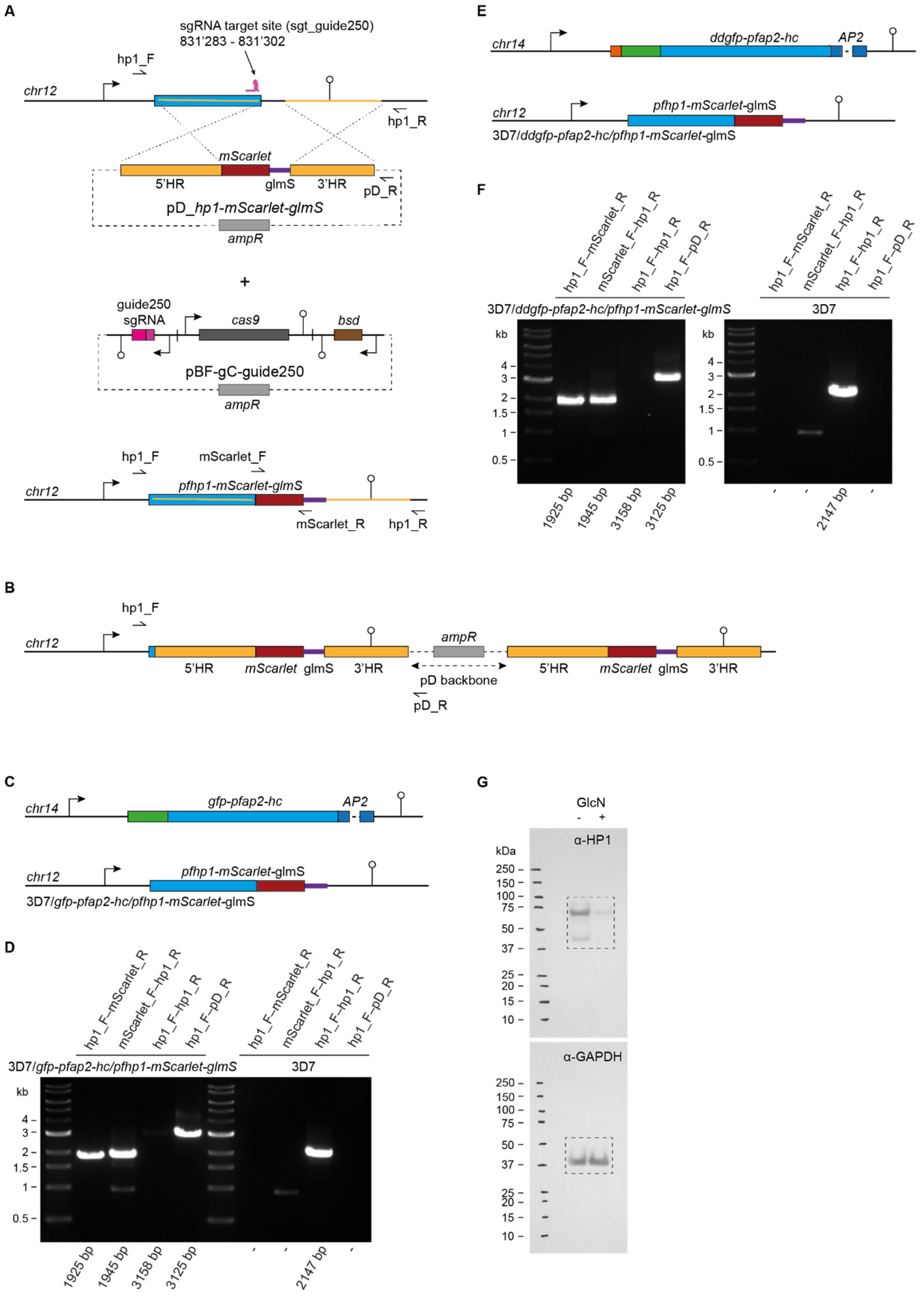

Figure S6. Generation of the 3D7/GFP-PfAP2-HC/PfHP1-mScarlet-glmS and 3D7/DDGFP-PfAP2-HC/PfHP1-mScarlet-glmS parasite lines, Related to Figures 5 and 6
(A) Schematic maps of the wild-type *pfhp1* locus (PF3D7_1220900) in 3D7/GFP-PfAP2-HC and 3D7/DDGFP-PfAP2-HC parasites (top), the CRISPR/Cas9 transfection vectors pD_*hp1-mScarlet-glmS* and pBF-gC-guide250 (Bui et al., 2019) (centre), and the modified *pfhp1* locus after

CRISPR/Cas9-based genome editing (bottom). The position of the sgt_guide250 sgRNA target sequence is indicated (chromosome 12 coordinates). The pD_*hp1-mScarlet-glmS* donor plasmid contains the *mScarlet* sequence (*red*) followed by the *glmS* ribozyme sequence (purple) flanked by homology regions (HR, yellow) for homology-directed repair. The pBF-gC-guide250 plasmid (Bui et al., 2019) contains expression cassettes for SpCas9 (dark grey), the sgRNA (pink) and the blasticidin deaminase (*bsd*) resistance marker (brown). Successful gene editing results in the expression of a C-terminally tagged PfHP1-mScarlet protein controlled by the *glmS* ribozyme element. PCR primer binding sites are indicated by half arrows and were used to confirm successful gene editing.
(B) Schematic map of the modified *pfhp1* locus after CRISPR/Cas9-based genome editing in the event of donor plasmid concatemer integration into the genome. PCR primer binding sites are indicated by arrows and were used to check for donor plasmid concatemer integration.
(C) Schematic maps of the *gfp-pfap2-hc* locus (top, see Figure S1) and the *pfhp1-mScarlet-glmS* locus (bottom) in successfully edited 3D7/GFP-PfAP2-HC/PfHP1-mScarlet-glmS parasites.
(D) PCR on gDNA from 3D7/GFP-PfAP2-HC/PfHP1-mScarlet-glmS and 3D7 wild-type parasites. Primers hp1_F and hp1_R bind to chromosomal sequences outside the HRs and amplify a 3158 bp or 2147 bp fragment from the edited or wild-type *pfhp1* locus, respectively. The hp1_F-mScarlet_R and mScarlet_F-hp1_R primer combinations are specific for the edited locus and amplify 1925 bp and 1945 bp fragments, respectively. Primer pD_R binds to the donor plasmid backbone and, when used in combination with primer hp1_F, will amplify a fragment of 3125 bp if a donor plasmid concatemer was integrated into the genome.
(E) Schematic maps of the *ddgfp-pfap2-hc* locus (top, see Figure S2) and the *pfhp1-mScarlet-glmS* locus (bottom) in successfully edited 3D7/DDGFP-PfAP2-HC/PfHP1-mScarlet-glmS parasites.
(F) PCR on gDNA from 3D7/DDGFP-PfAP2-HC/PfHP1-mScarlet-glmS and 3D7 wild-type parasites. Primer explanations are as in panel D. Primer combination mScarlet_F-hp1_R results in a faint non-specific product at ~1000 bp in all reactions (panels D and F).
(G) Full sized Western blot of the sections shown in Figure 5C showing PfHP1-mScarlet expression levels in 3D7/GFP-PfAP2-HC/PfHP1-mScarlet-glmS parasites grown in the absence (–) or presence (+) of GlcN. The membrane was first probed with α-PfHP1 antibodies (top) before inactivation of horseradish peroxidase with 2 mM NaN_3, followed by re-probing with the α-GAPDH antibodies (bottom) used as a loading control. Dashed boxes show the sections presented in Figure 5C.

A

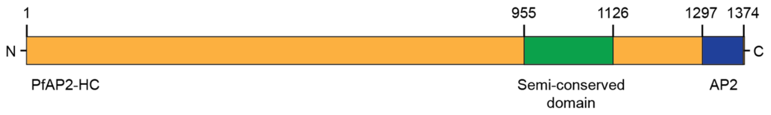

B

1 – 1374: Full length, % sequence identity								
	**Pv**	**Pk**	**Poc**	**Pm**	**Pb**	**Py**	**Pc**	**Pf**
**Pv**	100	81	46	46	37	38	37	35
**Pk**	81	100	47	46	37	37	36	35
**Poc**	46	47	100	46	37	38	38	31
**Pm**	46	46	46	100	40	41	40	34
**Pb**	37	37	37	40	100	91	83	32
**Py**	38	37	38	41	91	100	85	33
**Pc**	37	36	38	40	83	85	100	31
**Pf**	35	35	31	34	32	33	31	100

C

955 – 1126: Semi-conserved domain, % sequence identity								
	**Pm**	**Poc**	**Pv**	**Pk**	**Pf**	**Pb**	**Py**	**Pc**
**Pm**	100	78	77	76	64	62	64	62
**Poc**	78	100	76	75	67	59	60	60
**Pv**	77	76	100	95	66	58	60	59
**Pk**	76	75	95	100	66	59	60	59
**Pf**	64	67	66	66	100	53	56	55
**Pb**	62	59	58	59	53	100	95	92
**Py**	64	60	60	60	56	95	100	95
**Pc**	62	60	59	59	55	92	95	100

D

1297 – 1374: AP2 domain, % sequence identity								
	**Pb**	**Pc**	**Py**	**Pm**	**Poc**	**Pv**	**Pk**	**Pf**
**Pb**	100	99	99	94	94	95	95	90
**Pc**	99	100	100	95	95	96	96	91
**Py**	99	100	100	95	95	96	96	91
**Pm**	94	95	95	100	97	99	99	94
**Poc**	94	95	95	97	100	96	96	94
**Pv**	95	96	96	99	96	100	100	95
**Pk**	95	96	96	99	96	100	100	95
**Pf**	90	91	91	94	94	95	95	100

Figure S7. AP2-HC amino acid sequence comparison between orthologs of different *Plasmodium* species, Related to Figures 4 and 5
(A) Schematic map of the PfAP2-HC protein showing the location of the AP2 domain (blue) and a semi-conserved domain (green) identified via a multiple sequence alignment of AP2-HC orthologues from *P. vivax* (PVX_117665), *P. knowlesi* (PKNH_1225800), *P. malariae* (PmUG01_12060900), *P. ovale curtisi* (PocGH01_12058800), *P. berghei* (PBANKA_1319700), *P. yoelii* (PY17X_1323500) and *P. chabaudi* (PCHAS_1323000). Numbers refer to the amino acid position within the PfAP2-HC sequence.
(B, C, D) Amino acid sequence identity matrices of AP2-HC orthologues from eight *Plasmodium* species, comparing the full length protein (panel B), a semi-conserved domain of 172 amino acids (panel C), and the AP2 domain (panel D). Pf, *P. falciparum*. Pv, *P. vivax*. Pk, *P. knowlesi*. Pm, *P. malariae*. Poc, *P. ovale curtisi*. Pb, *P. berghei*. Py, *P. yoelii*. Pc, *P. chabaudi*.

Table S1. Oligonucleotide sequences used for cloning of CRISPR/Cas9 transfection vectors, Related to Figures 1, 2, 4, 5 and 6

Oligonucleotide name	Oligonucleotide sequence 5'→3'	Plasmid name	Cell line name
PCRA_F (Filarsky et al., 2018)	ctggcgtaatagcgaagagg	pD_*gfp-pfap2-hc*, pD_*gfp-pfap2-hc-ΔDBD*	3D7/GFP-PfAP2-HC, 3D7/GFP-PfAP2-HC-ΔDBD
PCRA_R (Filarsky et al., 2018)	cattaatgaatcggccaacg	pD_*gfp-pfap2-hc*, pD_*gfp-pfap2-hc-ΔDBD*	3D7/GFP-PfAP2-HC, 3D7/GFP-PfAP2-HC-ΔDBD
ap2-hc-5'_HR1_F	CGTTGGCCGATTCATTAATGcttatattgtattcagttgattctaac	pD_*gfp-pfap2-hc*	3D7/GFP-PfAP2-HC
ap2-hc-5'_HR1_R	TTCTCCTTTACTCATattttattcttattttgtgtattggttataag	pD_*gfp-pfap2-hc*	3D7/GFP-PfAP2-HC
ap2-hc-5'_GFP_F	AATAAGAATAAAATatgagtaaaggagaagaacttttcac	pD_*gfp-pfap2-hc*	3D7/GFP-PfAP2-HC
ap2-hc-5'_GFP_R	ACTGAATATTCATTtttgtatagttcatccatgccatg	pD_*gfp-pfap2-hc*	3D7/GFP-PfAP2-HC
ap2-hc-5'_HR2_re_F	TGAACTATACAAAaatgaatattcagtttataataagcc	pD_*gfp-pfap2-hc*	3D7/GFP-PfAP2-HC
ap2-hc-5'_HR2_re_R	CCTCTTCGCTATTACGCCAGggtcatctaaatttccattagg	pD_*gfp-pfap2-hc*	3D7/GFP-PfAP2-HC
sgRNA_ap2-hc-5'-1_F	*TATT*gaaacacataacgagcttaa	pH_gC-*ap2-hc-5'*-1	3D7/GFP-PfAP2-HC
sgRNA_ap2-hc-5'-1_R	*AAAC*ttaagctcgttatgtgtttc	pH_gC-*ap2-hc-5'*-1	3D7/GFP-PfAP2-HC
ap2-hc-5'_HR3_F	CGAGTCAGTGAGCGAGGActtatatttgtattcagttgattctaac	pFDon_*ddgfp-pfap2-hc*	3D7/DDGFP-PfAP2-HC
ap2-hc-5'_HR3_R	GTTTCCACCTGCACTCCCATattttattcttattttgtgtattggttataag	pFDon_*ddgfp-pfap2-hc*	3D7/DDGFP-PfAP2-HC
ap2-hc-5'_DD_F	ACACAAAATAAGAATAAAATatgggagtgcaggtggaaac	pFDon_*ddgfp-pfap2-hc*	3D7/DDGFP-PfAP2-HC
ap2-hc-5'_DD_R	TTCTTCTCCTTTACTCATACTAGAACCGGTttccagttttagaagctccacac	pFDon_*ddgfp-pfap2-hc*	3D7/DDGFP-PfAP2-HC
ap2-hc-5'_HR4_F	GAGCTTCTAAAACTGGAAACCGGTTCTAGTatgagtaaaggagaagaacttttcac	pFDon_*ddgfp-pfap2-hc*	3D7/DDGFP-PfAP2-HC
ap2-hc-5'_HR4_R	CTTTTCTCTTGTGGATCCGggtcatctaaatttccattagg	pFDon_*ddgfp-pfap2-hc*	3D7/DDGFP-PfAP2-HC
sgRNA_ap2-hc-5'-2_F	*TATT*taacaatatttctgtatcta	pH_gC-*ap2-hc-5'*-2	3D7/DDGFP-PfAP2-HC
sgRNA_ap2-hc-5'-2_R	*AAAC*tagatacagaaatattgtta	pH_gC-*ap2-hc-5'*-2	3D7/DDGFP-PfAP2-HC
ap2-hc-3'_HR1_re_F	CGTTGGCCGATTCATTAATGtaatagagatgaaaatagacaggca	pD_*gfp-pfap2-hc-ΔDBD*	3D7/GFP-PfAP2-HC-ΔDBD
ap2-hc-3'_HR1_re_R	acttttatcataatatactcctttTTAggcttcaaatgatgaaataaccc	pD_*gfp-pfap2-hc-ΔDBD*	3D7/GFP-PfAP2-HC-ΔDBD
ap2-hc-3'_HR2_re_F	ggttatttcatcatttgaagccTAAaaaggagtatattatgataaaagtag	pD_*gfp-pfap2-hc-ΔDBD*	3D7/GFP-PfAP2-HC-ΔDBD
ap2-hc-3'_HR2_re_R	aacaataattcagcttctttttctctctccattctattgctttttgtc	pD_*gfp-pfap2-hc-ΔDBD*	3D7/GFP-PfAP2-HC-ΔDBD
ap2-hc-3'_HR3_F	acaaaaagcaatagaatggagagagaaaaaagaagctgaattattgtt	pD_*gfp-pfap2-hc-ΔDBD*	3D7/GFP-PfAP2-HC-ΔDBD
ap2-hc-3'_HR3_R	CCTCTTCGCTATTACGCCAGatcatatccatctccatacacaaatg	pD_*gfp-pfap2-hc-ΔDBD*	3D7/GFP-PfAP2-HC-ΔDBD
sgRNA_ap2-hc-3'_F	*TATT*ctagacaaaaggctattgaa	pH_gC-*ap2-hc-3'*	3D7/GFP-PfAP2-HC-ΔDBD
sgRNA_ap2-hc-3'_R	*AAAC*ttcaatagccttttgtctag	pH_gC-*ap2-hc-3'*	3D7/GFP-PfAP2-HC-ΔDBD
ap2-hc-KO_HR1_F	TCAGGGTAGCTGATATCGGATCCcacataacgagcttaatgg	p_gCH-*pfap2-hc*-KO	3D7/PfAP2-HC-KO
ap2-hc-KO_HR1_R	CCTTTTCTCTTGTcattatattcttcaatgtctattac	p_gCH-*pfap2-hc*-KO	3D7/PfAP2-HC-KO
ap2-hc-KO_hDHFR_F	AAGAATATAATGacaagagaaaaggcagaaac	p_gCH-*pfap2-hc*-KO	3D7/PfAP2-HC-KO
ap2-hc-KO_hDHFR_R	CATTACACAAGGACtttaataaatatgttcttatatataatgag	p_gCH-*pfap2-hc*-KO	3D7/PfAP2-HC-KO
ap2-hc-KO_HR2_F	CATATTTATTAAAgtccttgtgtaatgaaaatatc	p_gCH-*pfap2-hc*-KO	3D7/PfAP2-HC-KO
ap2-hc-KO_HR2_R	GAGCGAGGAAGCGGAAGCTTgtgtacttggttgatcatatag	p_gCH-*pfap2-hc*-KO	3D7/PfAP2-HC-KO
sgRNA_ap2-hc-KO_F	*TATT*cgttgtactagtaacattgg	p_gCH-*pfap2-hc*-KO	3D7/PfAP2-HC-KO
sgRNA_ap2-hc-KO_R	*AAAC*ccaatgttactagtacaacg	p_gCH-*pfap2-hc*-KO	3D7/PfAP2-HC-KO
F158 (Bui et al., 2019)	CGTTGGCCGATTCATTAATGaaaggatattcagatgatgag	pD_*hp1-mScarlet-glmS*	3D7/GFP-PfAP2-HC/PfHP1-mScarlet-glmS, 3D7/DDGFP-PfAP2-HC/PfHP1-mScarlet-glmS
hp1_HR1_R	CCTTTACTACCTGCGGATCCcgctgttctatatcttaatc	pD_*hp1-mScarlet-glmS*	3D7/GFP-PfAP2-HC/PfHP1-mScarlet-glmS,

			3D7/DDGFP-PfAP2-HC/PfHP1-mScarlet-glmS
hp1_mScarlet_F	GATTAAGATATAGAACAGCGggatccgcaggtagtaaagg	pD_*hp1-mScarlet-glmS*	3D7/GFP-PfAP2-HC/PfHP1-mScarlet-glmS, 3D7/DDGFP-PfAP2-HC/PfHP1-mScarlet-glmS
hp1_mScarlet_R	TTGAGAAAATAAGAACAAGAtcatttatataattcatccattcc	pD_*hp1-mScarlet-glmS*	3D7/GFP-PfAP2-HC/PfHP1-mScarlet-glmS, 3D7/DDGFP-PfAP2-HC/PfHP1-mScarlet-glmS
hp1_glmS_F	GAATGGATGAATTATATAAATGAtcttgttcttattttctcaatagg	pD_*hp1-mScarlet-glmS*	3D7/GFP-PfAP2-HC/PfHP1-mScarlet-glmS, 3D7/DDGFP-PfAP2-HC/PfHP1-mScarlet-glmS
hp1_glmS_R	TGTATATTTGCATAATAAAAatttttcttcctcctaagattgtaaaag	pD_*hp1-mScarlet-glmS*	3D7/GFP-PfAP2-HC/PfHP1-mScarlet-glmS, 3D7/DDGFP-PfAP2-HC/PfHP1-mScarlet-glmS
hp1_HR2_F	ATCTTAGGAGGAAGAAAAATttttattatgcaaatatacatatatac	pD_*hp1-mScarlet-glmS*	3D7/GFP-PfAP2-HC/PfHP1-mScarlet-glmS, 3D7/DDGFP-PfAP2-HC/PfHP1-mScarlet-glmS
R163 (Bui et al., 2019)	CCTCTTCGCTATTACGCCAGgaggttaaaattctaaactatatg	pD_*hp1-mScarlet-glmS*	3D7/GFP-PfAP2-HC/PfHP1-mScarlet-glmS, 3D7/DDGFP-PfAP2-HC/PfHP1-mScarlet-glmS

Supplemental Table 1. Oligonucleotide sequences used for cloning of CRISPR/Cas9 transfection vectors. Oligonucleotide names and sequences are shown alongside the plasmid and parasite cell lines they were used to generate. Oligonucleotide sequences used to generate PCR fragments for Gibson assembly reactions (Gibson overhangs) are in upper case. Oligonucleotide sequences required for ligation of annealed double-stranded sgRNA-encoding sequences into the *Bsa*I site of the sgRNA expression cassette are italicized in upper case. A premature STOP codon is highlighted in red font.

Table S2. Primers used for PCRs on gDNA of CRISPR/Cas9-edited gene loci, Related to Figures 1, 2, 4, 5 and 6

Primer name	Primer sequence 5' → 3'	Cell line name
pD_F	accgcctttgagtgagc	
pD_R	cgaaaagtgccacctgacg	
ap2-hc-5'_F	attacttatattttattttctcttcaaagaaa	3D7/GFP-PfAP2-HC, 3D7/DDGFP-PfAP2-HC
gfp_R	tccagtgaaaagttcttctcct	3D7/GFP-PfAP2-HC, 3D7/DDGFP-PfAP2-HC
gfp_F	acatggcatggatgaactatacaaa	3D7/GFP-PfAP2-HC, 3D7/DDGFP-PfAP2-HC
ap2-hc-5'_R	acacaaacgctttcactatcttct	3D7/GFP-PfAP2-HC, 3D7/DDGFP-PfAP2-HC
ap2-hc-KO_F	ctaacaatatttctgtatctaaggg	3D7/PfAP2-HC-KO
hDHFR_R	aacgatgcagtttagcgaacc	3D7/PfAP2-HC-KO
hDHFR_F	atgtccaggaggagaaagg	3D7/PfAP2-HC-KO
ap2-hc-KO_R	aggttattttttaactgattatttagagg	3D7/PfAP2-HC-KO
ap2-hc_F	attaaagaattttggagttcctcc	3D7/PfAP2-HC-KO
ap2-hc_R	ctttgtgcattcatcctcagg	3D7/PfAP2-HC-KO
ap2-hc-3'_F	aataaccttcagaagaaatcgcaaa	3D7/GFP-PfAP2-HC-ΔDBD
ap2-hc-3'_R	atcggatatttattctcttgctgttg	3D7/GFP-PfAP2-HC-ΔDBD
hp1_F	gtgtgtgtttaagaaaaaatatg	3D7/GFP-PfAP2-HC/PfHP1-mScarlet-glmS, 3D7/DDGFP-PfAP2-HC/PfHP1-mScarlet-glmS
mScarlet_R	tgtatatttgcataataaaatcatttatataattcatccattccacc	3D7/GFP-PfAP2-HC/PfHP1-mScarlet-glmS, 3D7/DDGFP-PfAP2-HC/PfHP1-mScarlet-glmS
mScarlet_F	gattaagatatagaacagcgggatccgcaggtagtaaagg	3D7/GFP-PfAP2-HC/PfHP1-mScarlet-glmS, 3D7/DDGFP-PfAP2-HC/PfHP1-mScarlet-glmS
hp1_R	catgtagccaaaatatgtg	3D7/GFP-PfAP2-HC/PfHP1-mScarlet-glmS, 3D7/DDGFP-PfAP2-HC/PfHP1-mScarlet-glmS

Supplemental Table 2. Primers used for PCRs on gDNA of CRISPR/Cas9-edited gene loci. Primer names and sequences are shown alongside the parasite cell lines from which gDNA was extracted to carry out PCRs to confirm successful gene editing.

TRANSPARENT METHODS

Parasite culture

P. falciparum 3D7 parasites were cultured as described (Trager and Jenson, 1978) in RPMI Medium 1640 [+] L-Glutamine (Life Technologies) supplemented with 25 mM HEPES, pH 6.72, 100 mM hypoxanthine, 24 mM sodium bicarbonate and 0.5% Albumax II. 2 mM choline chloride was added to the medium to reduce sexual commitment rates (Brancucci et al., 2017). Synchronisation of parasite growth was achieved by repeated sorbitol treatments of ring stage parasites (Lambros and Vanderberg, 1979). Parasite cultures were kept at 37 ºC under a gaseous mixture of 4% CO_2, 3% O_2 and 93% N_2.

Transfection constructs

Transgenic cell lines were generated by CRISPR/Cas9-based genome editing using a set of plasmids recently described (Filarsky et al., 2018). All sgRNA target sequences were identified using CHOPCHOP (Labun et al., 2016; Labun et al., 2019; Montague et al., 2014). 3D7/GFP-PfAP2-HC parasites were created using a two-plasmid approach, consisting of a CRISPR/Cas9 transfection vector pH_gC-*ap2-hc-5'*-1 and the donor plasmid pD_*gfp-pfap2-hc*. The pH_gC-*ap2-hc-5'*-1 plasmid was created by annealing complementary oligonucleotides (sgRNA_ap2-hc-5'-1_F and sgRNA_ap2-hc-5'-1_R) encoding the sgRNA target sequence sgt_ap2-hc-5'-1 (gaaacacataacgagcttaa; positioned at bps +124 to +143 of the *pfap2-hc* coding sequence) and ligating them into the *Bsa*I-digested pH-gC plasmid (Filarsky et al., 2018). The pD_*gfp-pfap2-hc* donor plasmid was produced by Gibson assembly (Gibson et al., 2010; Gibson et al., 2009) of four PCR products encoding (1) the plasmid backbone amplified from pUC19 using primers PCRA_F and PCRA_R (Filarsky et al., 2018), (2) a 5' homology region (HR) spanning 575 bp of the *pfap2-hc* upstream region amplified from 3D7 gDNA using primers ap2-hc-5'_HR1_F and ap2-hc-5'_HR1_R, (3) the *gfp* coding sequence amplified from plasmid pD_*ap2g-gfp-dd-glmS* (Filarsky et al., 2018) using primers ap2-hc-5'_GFP_F and ap2-hc-5'_GFP_R, and (4) a 745 bp 3' HR corresponding to the *pfap2-hc* coding region +3 to +748. Fragment 4 was ordered as synthetic sequence (GenScript) with the first 274 bp recodonised and was amplified from plasmid pUC57-re-*ap2-hc*-1 using primers ap2-hc-5'_HR2_re_F and ap2-hc-5'_HR2_re_R.
The 3D7/DDGFP-PfAP2-HC parasite line was generated using plasmids pH_gC-*ap2-hc-5'*-2 and pFDon_*ddgfp-pfap2-hc*. To generate pH_gC-*ap2-hc-5'*-2, complementary oligonucleotides (sgRNA_ap2-hc-5'-2_F and sgRNA_ap2-hc-5'-2_R) encoding the sgRNA target sequence sgt_ap2-hc-5'-2 (taacaatatttctgtatcta; positioned at bps +36 to +55 of the *pfap2-hc* coding sequence) were annealed and ligated into the *Bsa*I-digested pH-gC plasmid (Filarsky et al., 2018). pFDon_*ddgfp-pfap2-hc* donor plasmid was created by Gibson assembly of four fragments: (1) the pFDon plasmid (Filarsky et al., 2018) digested with *Hind*III and *Eco*RI, (2, 3) the 5' HR and the *gfp*-3'HR were amplified from plasmid pD_*gfp-pfap2-hc* (described above) with primers ap2-hc-5'_HR3_F and ap2-hc-5'_HR3_R, and ap2-hc-5'_HR4_F and ap2-hc-5'_HR4_R, respectively. The final fragment 4 encoding the FKBP destabilizing domain (*dd*) sequence (plus C-terminal TGSS linker) was amplified from pD_*ap2g-gfp-dd-glmS* (Filarsky et al., 2018) using primers ap2-hc-5'_DD_F and ap2-hc-5'_DD_R.
Parasite line 3D7/GFP-PfAP2-HC-ΔDBD was created by CRISPR/Cas9 editing of 3D7/GFP-PfAP2-HC parasites using plasmids pH_gC-*ap2-hc-3'* and pD_*gfp-pfap2-hc-ΔDBD*. The sgRNA-encoding oligonucleotides sgRNA_ap2-hc-3'_F and sgRNA_ap2-hc-3'_R were annealed and ligated into the *Bsa*I-digested pH-gC plasmid (Filarsky et al., 2018), as above, to create pH_gC-*ap2-hc-3'*. The sgRNA target sequence sgt_ap2-hc-3' (ctagacaaaaggctattgaa) is positioned at bps +4070 to +4089 of the *pfap2-hc* coding sequence. To create pD_*gfp-pfap2-hc-ΔDBD*, a synthetic DNA sequence (GenScript), corresponding to the *pfap2-hc* coding sequence +3528 to +4125 with the intron removed and the sequence +3954 to +4125 recodonised (plasmid pUC57-re-*ap2-hc*-2). To introduce a STOP codon prior to the sequence encoding the AP2 DBD two overlapping PCR fragments (1, 2) were amplified from pUC57-re-*ap2-hc*-2 using primers ap2-hc-3'_HR1_re_F and ap2-hc-3'_HR1_re_R, and ap2-hc-3'_HR2_re_F and ap2-hc-3'_HR2_re_R, respectively. The ap2-hc-3'_HR1_re_R and ap2-hc-3'_HR2_re_F primers introduce a TAA STOP codon at amino acid position 1319 (R1319*). Fragments (1, 2) were assembled together with fragment 3 representing the plasmid backbone amplified from pUC19 using primers PCRA_F and PCRA_R (Filarsky et al., 2018), and fragment 4 representing the 3' HR beginning at nucleotide +4088 of the *pfap2-hc* coding sequence and ending 852 bp downstream of the native STOP codon (76 bp into the neighbouring gene PF3D7_1456100) and amplified from 3D7 gDNA using primers ap2-hc-3'_HR3_F and ap2-hc-3'_HR3_R.
3D7/GFP-PfAP2-HC/PfHP1-mScarlet-glmS and 3D7/DDGFP-PfAP2-HC/PfHP1-mScarlet-glmS parasite lines were generated by editing the endogenous *pfhp1* locus in parasites lines 3D7/GFP-PfAP2-HC and 3D7/DDGFP-PfAP2-HC, respectively. The recently published CRISPR/Cas9 plasmid

pBF-gC-guide250 (Bui et al., 2019) was used in combination with the donor plasmid pD_*hp1-mScarlet-glmS*. The donor construct was created by joining five fragments in a Gibson assembly consisting of (1, 2) previously described 5' and 3' HRs amplified from plasmid pD-PfHP1-KO (Bui et al., 2019), using primers F158 and hp1_HR1_R, and hp1_HR2_F and R163, respectively. Fragment 3, consisting of a *P. falciparum* codon-optimised *mScarlet* sequence with an N-terminal GSAG linker, was amplified from the plasmid pD_*ap2-g-mScarlet* (Brancucci et al., manuscript in preparation) using primers hp1_mScarlet_F and hp1_mScarlet_R. The *glmS* sequence (fragment 4) was amplified from plasmid pL6-3HA_glmS-246 (kind gift from Dave Richard) using primers hp1_glmS_F and hp1_glmS_R, and finally, the plasmid backbone (fragment 5), was amplified from pUC19 with primers PCRA_F and PCRA_R (Filarsky et al., 2018).
The 3D7/PfAP2-HC-KO cell line was created using a single plasmid CRISPR/Cas9 approach. The mother plasmid p_gC (Filarsky et al., 2018) formed the backbone to create p_gCH-*pfap2-hc*-KO. p_gC was digested with *Bam*HI and *Hind*III and used in a Gibson assembly with (1) a 5' HR spanning bps +128 to +533 of the *pfap2-hc* coding sequence, amplified from 3D7 gDNA with primers ap2-hc-KO_HR1_F and ap2-hc-KO_HR1_R, (2) a *hdhfr* expression cassette amplified from plasmid p_gCH-*gdv1*-asKO (Filarsky et al., 2018) (primers ap2-hc-KO_hDHFR_F and ap2-hc-KO_hDHFR_R), and (3) a 3' HR spanning bps +3042 to +3460 of the *pfap2-hc* coding sequence, amplified from 3D7 gDNA using primers ap2-hc-KO_HR2_F and ap2-hc-KO_HR2_R. The resulting plasmid, p_gCH-*pfap2-hc*-KO-pre, was digested with *Bsa*I and the sgRNA-encoding sequence sgt_ap2-hc-KO (cgttgtactagtaacattgg; position +1724 to +1743 of the *pfap2-hc* coding sequence, negative strand) was created by annealing the complementary oligonucleotides sgRNA_ap2-hc-KO_F and sgRNA_ap2-hc-KO_R, and ligated into the *Bsa*I site creating the final p_gCH-*pfap2-hc*-KO plasmid.
Oligonucleotide sequences used in cloning are provided in Table S1.

Transfection and transgenic cell lines

P. falciparum parasite transfections were carried out as described (Filarsky et al., 2018; Voss et al., 2006). A total of 100 µg plasmid DNA (two-plasmid CRISPR/Cas9 approach: 50 µg of each plasmid; single-plasmid CRISPR/Cas9 approach: 100 µg plasmid) was transfected into 3D7/WT or previously engineered parasites and the cultures allowed to recover for 24 hours by growth in drug-free culture medium. Selection of transgenics was then initiated by the addition of 4 nM WR99210 for a total of six days for pH-derived plasmids, or continuously for plasmid p_gCH-*pfap2-hc*-KO. Parasites transfected with the pBF-gC-guide250 construct were selected with 2.5 µg/mL blasticidin-S-hydrochloride for a total of ten days. 3D7/DDGFP-PfAP2-HC parasites were cultured in the presence of 700 nM Shield-1 (+Shield-1) unless otherwise stated. Induction of PfHP1 depletion in parasite lines 3D7/GFP-PfAP2-HC/PfHP1-mScarlet-glmS and 3D7/DDGFP-PfAP2-HC/PfHP1-mScarlet-glmS was achieved by the addition of 2.5 mM glucosamine (GlcN, Sigma #G4875). Limiting dilution cloning was carried out on parasites lines 3D7/GFP-PfAP2-HC, 3D7/DDGFP-PfAP2-HC and 3D7/GFP-PfAP2-HC-ΔDBD as described (Thomas et al., 2016). Successful gene editing was confirmed by PCR on gDNA using primers listed in Table S2.

Fluorescence microscopy

Live cell fluorescence imaging was performed as previously described (Witmer et al., 2012) with the minor modification of nuclear staining with Hoechst (Merck) instead of DAPI at a final concentration of 5 µg/ml. IFAs were carried out on methanol-fixed cells using primary antibodies mouse mAb α-GFP (Roche Diagnostics #11814460001) (1:100) and rabbit α-PfHP1 (Brancucci et al., 2014) (1:100). Secondary antibodies Alexa Fluor 488-conjugated α-mouse IgG (Invitrogen #A11001) and Alexa Fluor 568-conjugated α-rabbit IgG (Invitrogen #A11011) were used, each at 1:250 dilution. Nuclei were stained during slide preparation with Vectashield containing DAPI (Vector Laboratories). Images were acquired on a Leica DM 5000B microscope with a Leica DFC 345 FX camera using the Leica application suite (LAS) software. Image processing was carried out using Fiji (Schindelin et al., 2012). For each experiment, all images were acquired and processed with identical settings.

Western blot

Whole parasite protein extracts were prepared by first releasing parasites from the iRBC by saponin lysis (0.15% in PBS) followed by suspension of the parasite pellet in UREA/SDS lysis buffer [(8 M Urea, 5% SDS, 50 mM Bis-Tris, 2 mM EDTA, 25 mM HCl, pH 6.5, 1 mM DTT, 1x protease inhibitor (Roche)] and separated on a NuPage 3-8% Tris-Acetate gel (Novex) using NuPage MES SDS Running Buffer (Novex). Proteins were detected with primary antibodies mouse mAb α-GFP (Roche Diagnostics #11814460001) (1:1000), rabbit α-PfHP1 (Brancucci et al., 2014) (1:5000), and mouse mAb α-GAPDH (Daubenberger et al., 2003), (1:10000), and secondary antibodies α-mouse IgG (H&L)-HRP (GE healthcare #NXA931) (1:5000) and α-rabbit IgG (H&L)-HRP (GE Healthcare #NA934)

(1:5000). Chemiluminescence signal was detected using KPL LumiGLO Reserve Chemiluminescent Substrate Kit (SeraCare #5430-0049).

Chromatin immunoprecipitations

Parasite cultures were synchronised to obtain an eight-hour growth window and harvested at peak PfAP2-HC expression at 36-44 hpi (Bartfai et al., 2010) from a 30 ml culture at 5% haematocrit and 4-5% parasitemia. Parasites were crosslinked with 1% formaldehyde for 10 min at 37 °C before quenching with 0.125 M glycine. The RBC membrane was lysed with 0.15% saponin and cytoplasmic lysis buffer [(CLB: 20 mM Hepes, 10 mM KCl, 1 mM EDTA, 1 mM EGTA, 0.65% NP-40, 1 mM DTT, 1x protease inhibitor (Roche)] was added to the parasite pellet to isolate nuclei. Nuclei were then snap-frozen in liquid nitrogen in CLB supplemented with 50% glycerol and stored at -80 °C. Chromatin isolation, shearing and immunoprecipitation was performed according to previously published protocols (Filarsky et al., 2018). To prepare chromatin, frozen nuclei were thawed, pelleted and resuspended in 150 µl sonication buffer [(50 mM Tris pH 8, 1% SDS, 10 mM EDTA, 1x protease inhibitor (Roche)], and were sonicated for 20 cycles of 30 sec ON/30 sec OFF (setting high, BioruptorTM Next Gen, Diagenode) to shear DNA to fragments of 100-600 bps. Fragment size was confirmed by de-crosslinking a 15 µl aliquot and visualising the purified DNA on a 2% agarose gel. ChIPs were performed by combining sonicated chromatin (500 ng DNA content) with either 1 µg mouse mAb α-GFP (Roche Diagnostics #11814460001) or 1 µg rabbit α-PfHP1 (Brancucci et al., 2014) in incubation buffer [(5% Triton-X-100, 750 mM NaCl, 5 mM EDTA, 2.5 mM EGTA, 100 mM Hepes pH 7.4, 0.2% bovine serum albumin, 1x protease inhibitor (Roche)] containing 10 µl protA and 10 µl protG Dynabeads (Life Technologies, #10008D and #10009D, respectively) in a total reaction volume of 300 µl. ChIP samples were incubated overnight at 4 °C with rotation. Beads were washed for 5 min at 4 °C, with rotation, with 400 µl wash buffers as follows: 2x wash buffer 1 (0.1% SDS, 0.1% DOC, 1% Triton-X100, 150 mM NaCl, 1 mM EDTA, 0.5 mM EGTA, 20 mM Hepes pH 7.4), 1x wash buffer 2 (0.1% SDS, 0.1% DOC, 1% Triton-X100, 500 mM NaCl, 1 mM EDTA, 0.5 mM EGTA, 20 mM Hepes pH 7.4), 1x wash buffer 3 (250 mM LiCl, 0.5% DOC, 0.5% NP-40, 1 mM EDTA, 0.5 mM EGTA, 20 mM Hepes pH 7.4), 2x wash buffer 4 (1 mM EDTA, 0.5 mM EGTA, 20 mM Hepes pH 7.4). Immunoprecipitated chromatin was eluted from the beads by shaking at room temperature for 20 min in 200 µl elution buffer (1% SDS, 0.1 M $NaHCO_3$) and de-crosslinked at 45 °C overnight in 1% SDS, 0.1 M $NaHCO_3$ and 1 M NaCl. Simultaneously, 30 µl of sonicated input chromatin was de-crosslinked under the same conditions. DNA was purified with QIAquick MinElute PCR columns (Qiagen). For each ChIP-seq experiment, twenty separate α-GFP ChIPs or four separate α-PfHP1 ChIPs were combined, with the exception of 3D7/GFP-PfAP2-HC/PfHP1-mScarlet-glmS α-PfHP1 ChIP-seq for which eight separate ChIPs were combined.

High throughput sequencing and data analysis

The obtained ChIPed DNA fragments were used to generate Illumina sequencing libraries according to Filarsky et al. (Filarsky et al., 2018). In brief, 1 ng of α-PfHP1 ChIP, α-GFP ChIP, or input DNA were end-repaired with T4 DNA polymerase (NEB, M0203L), Klenow DNA polymerase (NEB, M0210L), and T4 Polynucleotide Kinase (NEB, M0201L). The 3'ends of end-repaired DNA were extended with an A-overhang with 3' to 5' exonuclease-deficient Klenow DNA polymerase (NEB, M0212L). The resulting fragments were ligated to Nextflex 6bp adaptors (Bio Scientific, #514122) with the use of T4 DNA ligase (Promega, M1804). The libraries were amplified using an AT-rich optimized KAPA protocol using KAPA HiFi HotStart ready mix (KAPA Biosystems, KM2602), NextFlex primer mix (Bio Scientific, #514122) with the following PCR program: 98°C for 2 min; four cycles of 98°C for 20 sec, 62°C for 3 min; 62°C for 5 min. The fragments originating from mono-nucleosomes + 125 bp NextFlex adapter were selected using 2% E-Gel Size Select agarose gels (Invitrogen, #G6610-02) and amplified by PCR for nine cycles using the above conditions. Libraries were purified and adapter dimers removed with Agencourt AMPure XP beads purification using a 1:1 library:beads ratio (Beckman Coulter, #A63880). ChIP-seq libraries were sequenced on the Illumina NextSeq 500 system with a 20% phiX spike-in (Illumina, FC-110-3001) to generate 75 bp single-end reads (NextSeq 500/550 High Output v2 kit). The quality of the resulting reads were checked with FastQC (V0.11.8) and the reads were mapped against the *P. falciparum* 3D7 reference genome from PlasmoDB v26 (www.plasmodb.org) using BWA samse (v0.7.17-r1188) (Andrews, 2010; Li and Durbin, 2009). Mapped reads originating from the mitochondrial and apicoplast genome, multi-mapping reads, and reads having a mapping quality below 15 were removed (SAMtools v1.9) (Li et al., 2009) leaving between 4.8 and 25.4 million reads (note that replicate 2 of 3D7/GFP-PfAP2-HC/PfHP1-mScarlet-glmS cultured in presence of GlcN (Figure 5D) is based on 2.0 million reads). Before visualising the ChIP-seq data in the UCSC Genome browser (Figures 1E-G, 2D, 4C, 5D) or Signalmap (version 2.0.0.5) (Figure 1C), the libraries were normalised to the total amount of reads, reads per million, with bedtools genomeCoverageBed

(v2.27.1) and the enrichment over the input sample was calculated by dividing ChIP sample with input sample read counts, which subsequently was log2 transformed (one pseudo count was added to avoid division by zero) (Kent et al., 2002; Quinlan and Hall, 2010). Within the UCSC genome browser, tracks were smoothened (8) and the windowing function was set as 'mean'. To enable the visualisation of all chromosomes together Signalmap (version 2.0.0.5) was used. The average log2 Chip-over-input value was calculated in sequential 1kb windows to smoothen and compress the data set. PfHP1 values are visualised on the positive scale shifted up by 2 and PfAP2-HC (GFP) values are visualised in the negative scale shifted up by 1. The enrichment tracks were shifted to show the complete tracks. To show the genome-wide colocalization of PfHP1 and PfAP2-HC occupancy and comparison between cell lines the average log2 Chip-over-input ratios at coding genes were calculated using bedtools genomeCoverageBed (v2.27.1) (Quinlan and Hall, 2010) (Dataset S1) and visualised with Excel 2016. Genes with PfHP1 log2 ChIP-over-input ratios of greater than or equal to zero were classified as heterochromatic genes. The position of putative AP2-HC binding sites (i.e. CACACA motifs) has been defined using the position weight matrix of the CACACA motif as described by Campbell et al. (Campbell et al., 2010) and searching the *P falciparum* genome for matching sequences (fdr<0.05) with the use of the gimme scan tool of GimmeMotifs (van Heeringen and Veenstra, 2011).

Flow cytometry

Synchronous 3D7/DDGFP-PfAP2-HC and 3D7/WT parasites were split at 0-8 hpi to 0.1% parasitaemia and cultured either in the presence of 700 nM Shield-1 (+ Shield-1) or absence of Shield-1 (- Shield-1) during the duration of the multiplication assay. Synchronous 3D7/PfAP2-HC-KO parasites at 0-8 hpi were diluted to 0.1% parasiteamia. After 24 hours (24-32 hpi) parasite DNA was stained with SYBR Green DNA stain (1: 10,000) (Invitrogen #S7563) for 30 min at 37 °C and the fluorescence intensity was measured using a MACS Quant Analyzer 10 (at least 200,000 RBCs were measured per sample) to determine the parasitaemia (day 1). Measurements were repeated on day 3 and day 5 at 24-32 hpi. Data were analysed using the FlowJo_v10.6.1 software. Gating was performed to remove debris smaller than cell size, to include only single measurement events and to separate uninfected from infected RBCs based on the SYBR Green intensity of an uninfected RBC control sample (the gates for 'cells', 'singlets' and 'parasites', respectively, are shown in Figure S2).

Quantification of sexual conversion rates

3D7/DDGFP-PfAP2-HC and 3D7/WT parasites were synchronised with sorbitol to a 6-hour time window and again in the next generation after RBC invasion at 0-6 hpi, after which each culture was split and one half was maintained in the presence of Shield-1 (+ Shield-1) and the other half was cultured in the absence of Shield-1 (- Shield-1). Eighteen hours later (18-24 hpi), cultures were split again (1-2% parasitaemia, 2.5% haematocrit) and grown in either minimal fatty acid (mFA) medium [RPMI Medium 1640 [+] L-Glutamine (Life Technologies) supplemented with 25 mM HEPES, pH 6.72, 100 mM hypoxanthine, 24 mM sodium bicarbonate, 0.39% fatty acid-free BSA (Sigma #A6003), 30 μM oleic acid and 30 μM palmitic acid], which induces sexual commitment, or mFA medium supplemented with 2 mM choline chloride (mFA+CC), which inhibits sexual commitment (Brancucci et al., 2017). After 24 hours (42-48 hpi), mFA or mFA+CC medium was replaced with the standard Albumax medium used throughout this study (see above). After invasion into new RBCs, 50 mM N-acetylglucosamine (GlcNAc) was added to the cultures at 24-30 hpi (day 1 of gametocytogenesis) to prevent asexual parasite multiplication (Fivelman et al., 2007; Ponnudurai et al., 1986). The resulting gametocyte cultures were maintained in the presence of GlcNAc for a further 3 days. Parasitaemia on day 1 of gametocytogenesis (mixture of asexuals and sexual ring stage parasites) and gametocytemia on day 4 of gametocytogenesis were counted and sexual conversion rates were calculated (gametocytaemia [%] / parasitaemia [%] * 100 = sexual conversion rate [%]).

Microarray Experiments and Data Analysis

3D7/DDGFP-PfAP2-HC parasites, continuously cultured in the presence of Shield-1, were synchronised with sorbitol to obtain an eight-hour growth window (16-24 hpi) and again in the next generation after RBC invasion at 0-8 hpi. The culture was then split at 8-16 hpi and one half was maintained in the presence of Shield-1 (+Shield-1) and the other half was cultured in the absence of Shield-1 (-Shield-1) to achieve DDGFP-PfAP2-HC depletion. The parasites proceeded through the IDC and were harvested for total RNA extraction in the subsequent generation at the following time points: TP1 (8-16 hpi), TP2 (16-24 hpi), TP3 (24-32 hpi), TP4 (32-40 hpi) and TP5 (40-48 hpi). RNA isolation and cDNA synthesis were performed as previously described (Bozdech et al., 2003). Cy5-labelled sample cDNAs were hybridised against a Cy3-labelled cDNA reference pool prepared from 3D7 wild-type parasites (Brancucci et al., 2014). Equal amounts of Cy5- and Cy3-labelled samples

were hybridised on a *P. falciparum* 8×15K Agilent gene expression microarray (GEO platform ID GPL15130) (Painter et al., 2013) for 16 hours at 65°C in an Agilent hybridisation oven (G2545A). Slides were scanned using the GenePix scanner 4000B and GenePix pro 6.0 software (Molecular Devices). The raw microarray data representing relative steady state mRNA abundance ratios between each test sample and the reference pool (Cy5/Cy3 log2 ratios) were subjected to lowess normalization and background filtering as implemented by the Acuity 4.0 program (Molecular Devices). Flagged features and features with either Cy3 or Cy5 intensities lower than two-fold the background were removed. Log2 ratios for multiple probes per gene were averaged and genes recognized by non-uniquely mapping probes were removed from the dataset. Transcripts showing expression values in at least four of the five samples harvested for each time course were included for downstream analysis to identify genes differentially expressed [mean fold change cut-off > 2; p-value cut-off 0.01 (paired two-tailed Student's t-test)] between control (+Shield-1) and DDGFP-AP2-HC-depleted (-Shield-1) parasites. The processed microarray dataset is listed in Dataset S2.

Induction of gametocytogenesis by conditional depletion of PfHP1

3D7/DDGFP-PfAP2-HC/PfHP1-mScarlet-glmS parasites were synchronised to obtain an eight-hour growth window and re-synchronised in the following cycle at 0-8 hpi (generation 1). The culture was split into two populations, one grown in the presence of Shield-1 and one in the absence of Shield-1. Both parasite populations were synchronised again at 0-8 hpi in the following cycle (generation 2) and 2.5 mM glucosamine (GlcN, Sigma #G4875) was added to induce PfHP1 depletion (+Shield-1/+GlcN and –Shield-1/+GlcN). Parasites progressed through generation 2 and in generation 3, GlcN was removed from both populations and parasites were cultured on serum medium (0.5% Albumax replaced with 10% human serum) containing 50 mM N-acetylglucosamine (GlcNAc) to prevent asexual parasite multiplication (Fivelman et al., 2007; Ponnudurai et al., 1986). Live cell fluorescence imaging was performed in stage II and stage V gametocytes to observe PfHP1-mScarlet signals between +Shield-1 (DDGFP-AP2-HC-expressing) and -Shield-1 (DDGFP-AP2-HC-depleted) parasites. The experimental design is depicted schematically in Figure 6B.

Multiple sequence alignment of AP2-HC orthologues

Orthologues of PfAP2-HC (PF3D7_1456000) were identified on www.plasmodb.org (Aurrecoechea et al., 2009) from *P. vivax* (PVX_117665), *P. knowlesi* (PKNH_1225800), *P. malariae* (PmUG01_12060900), *P. ovale curtisi* (PocGH01_12058800), *P. berghei* (PBANKA_1319700), *P. yoelii* (PY17X_1323500) and *P. chabaudi* (PCHAS_1323000) and their full length amino acid sequences were aligned using Clustal X2.1 (Larkin et al., 2007) multiple sequence alignment on default settings. A tree was generated using Clustal X2.1 (Larkin et al., 2007) on default settings and the resulting identity matrix was tabulated. The AP2 domains of each orthologue were aligned separately and an identity matrix generated. A semi-conserved domain (spanning amino acids 995-1126 of PfAP2-HC) was identified, aligned separately and an identity matrix generated.

SUPPLEMENTAL REFERENCES

Andrews, S., (2010). FastQC: A quality control tool for high throughput sequence data. Available online at: https://www.bioinformatics.babraham.ac.uk/projects/fastqc.

Bozdech, Z., Llinas, M., Pulliam, B.L., Wong, E.D., Zhu, J., and DeRisi, J.L. (2003). The transcriptome of the intraerythrocytic developmental cycle of Plasmodium falciparum. PLoS Biol. 1(1), E5.

Brancucci, N.M.B., Gerdt, J.P., Wang, C., De Niz, M., Philip, N., Adapa, S.R., Zhang, M., Hitz, E., Niederwieser, I., Boltryk, S.D., et al. (2017). Lysophosphatidylcholine Regulates Sexual Stage Differentiation in the Human Malaria Parasite Plasmodium falciparum. Cell. 171(7), 1532-1544 e1515.

Bui, H.T.N., Niederwieser, I., Bird, M.J., Dai, W., Brancucci, N.M.B., Moes, S., Jenoe, P., Lucet, I.S., Doerig, C., and Voss, T.S. (2019). Mapping and functional analysis of heterochromatin protein 1 phosphorylation in the malaria parasite Plasmodium falciparum. Scientific Reports. 9(1), 16720.

Daubenberger, C.A., Tisdale, E.J., Curcic, M., Diaz, D., Silvie, O., Mazier, D., Eling, W., Bohrmann, B., Matile, H., and Pluschke, G. (2003). The N'-terminal domain of glyceraldehyde-3-phosphate dehydrogenase of the apicomplexan Plasmodium falciparum mediates GTPase Rab2-dependent recruitment to membranes. Biol. Chem. 384(8), 1227-1237.

Gibson, D.G., Smith, H.O., Hutchison, C.A., 3rd, Venter, J.C., and Merryman, C. (2010). Chemical synthesis of the mouse mitochondrial genome. Nat. Methods. 7(11), 901-903.

Gibson, D.G., Young, L., Chuang, R.Y., Venter, J.C., Hutchison, C.A., III, and Smith, H.O. (2009). Enzymatic assembly of DNA molecules up to several hundred kilobases. Nat. Methods. 6(5), 343-345.

Kent, W.J., Sugnet, C.W., Furey, T.S., Roskin, K.M., Pringle, T.H., Zahler, A.M., and Haussler, D. (2002). The human genome browser at UCSC. Genome Res. 12(6), 996-1006.

Labun, K., Montague, T.G., Gagnon, J.A., Thyme, S.B., and Valen, E. (2016). CHOPCHOP v2: a web tool for the next generation of CRISPR genome engineering. Nucleic Acids Res. 44(W1), W272-W276.

Labun, K., Montague, T.G., Krause, M., Torres Cleuren, Y.N., Tjeldnes, H., and Valen, E. (2019). CHOPCHOP v3: expanding the CRISPR web toolbox beyond genome editing. Nucleic Acids Res. 47(W1), W171-W174.

Lambros, C., and Vanderberg, J.P. (1979). Synchronization of Plasmodium falciparum erythrocytic stages in culture. J. Parasitol. 65(3), 418-420.

Larkin, M.A., Blackshields, G., Brown, N.P., Chenna, R., McGettigan, P.A., McWilliam, H., Valentin, F., Wallace, I.M., Wilm, A., Lopez, R., et al. (2007). Clustal W and Clustal X version 2.0. Bioinformatics. 23(21), 2947-2948.

Li, H., and Durbin, R. (2009). Fast and accurate short read alignment with Burrows-Wheeler transform. Bioinformatics. 25(14), 1754-1760.

Li, H., Handsaker, B., Wysoker, A., Fennell, T., Ruan, J., Homer, N., Marth, G., Abecasis, G., Durbin, R., and Genome Project Data Processing, S. (2009). The Sequence Alignment/Map format and SAMtools. Bioinformatics. 25(16), 2078-2079.

Montague, T.G., Cruz, J.M., Gagnon, J.A., Church, G.M., and Valen, E. (2014). CHOPCHOP: a CRISPR/Cas9 and TALEN web tool for genome editing. Nucleic Acids Res. 42(Web Server issue), W401-W407.

Quinlan, A.R., and Hall, I.M. (2010). BEDTools: a flexible suite of utilities for comparing genomic features. Bioinformatics. 26(6), 841-842.

Schindelin, J., Arganda-Carreras, I., Frise, E., Kaynig, V., Longair, M., Pietzsch, T., Preibisch, S., Rueden, C., Saalfeld, S., Schmid, B., et al. (2012). Fiji: an open-source platform for biological-image analysis. Nat. Methods. 9(7), 676-682.

Thomas, J.A., Collins, C.R., Das, S., Hackett, F., Graindorge, A., Bell, D., Deu, E., and Blackman, M.J. (2016). Development and Application of a Simple Plaque Assay for the Human Malaria Parasite Plasmodium falciparum. PLoS. ONE. 11(6), e0157873.

Trager, W., and Jenson, J.B. (1978). Cultivation of malarial parasites. Nature. 273(5664), 621-622.

van Heeringen, S.J., and Veenstra, G.J. (2011). GimmeMotifs: a de novo motif prediction pipeline for ChIP-sequencing experiments. Bioinformatics. 27(2), 270-271.

Voss, T.S., Healer, J., Marty, A.J., Duffy, M.F., Thompson, J.K., Beeson, J.G., Reeder, J.C., Crabb, B.S., and Cowman, A.F. (2006). A var gene promoter controls allelic exclusion of virulence genes in Plasmodium falciparum malaria. Nature. 439(7079), 1004-1008.

Witmer, K., Schmid, C.D., Brancucci, N.M., Luah, Y.H., Preiser, P.R., Bozdech, Z., and Voss, T.S. (2012). Analysis of subtelomeric virulence gene families in Plasmodium falciparum by comparative transcriptional profiling. Mol. Microbiol. 84(2), 243-259.
